# IGF2BP3 redirects glycolytic flux to promote one-carbon metabolism and RNA methylation

**DOI:** 10.1016/j.celrep.2025.116330

**Published:** 2025-09-26

**Authors:** Gunjan Sharma, Martin Gutierrez, Anthony E. Jones, Shruti Kapoor, Amit Kumar Jaiswal, Zachary T. Neeb, Amy Rios, Poornima Dorairaj, Michelle L. Thaxton, Tasha L. Lin, Tiffany M. Tran, Lyna E.S. Kabbani, Alexander J. Ritter, Georgia M. Scherer, Jacob P. Sorrentino, Linsey Stiles, Johanna ten Hoeve, Robert D. Damoiseaux, Neil K. Garg, Ajit S. Divakaruni, Jeremy R. Sanford, Dinesh S. Rao

**Affiliations:** 1 Department of Pathology and Laboratory Medicine, University of California, Los Angeles, Los Angeles, CA, USA; 2 Department of Molecular, Cell and Developmental Biology and Center for Molecular Biology of RNA, University of California, Santa Cruz, Santa Cruz, CA, USA; 3 Department of Molecular and Medical Pharmacology, University of California, Los Angeles, Los Angeles, CA, USA; 4 Division of Hematology and Oncology, Department of Medicine, University of California, Los Angeles, Los Angeles, CA, USA; 5 Department of Medicine, University of California, Los Angeles, Los Angeles, CA, USA; 6 Department of Chemistry and Biochemistry, University of California, Los Angeles, Los Angeles, CA, USA; 7 UCLA Metabolomics Center, University of California, Los Angeles, Los Angeles, CA 90095, USA; 8 Center for Biomolecular Science & Engineering, University of California, Santa Cruz, Santa Cruz, CA, USA; 9 Jonsson Comprehensive Cancer Center, University of California, Los Angeles, Los Angeles, CA, USA; 10 Broad Stem Cell Research Center, University of California, Los Angeles, Los Angeles, CA, USA; 11 Lead contact

## Abstract

Insulin-like growth factor 2 mRNA-binding protein 3 (IGF2BP3), an oncofetal RNA-binding protein and a non-canonical reader of N6-methyladenosine (m^6^A) mRNA modifications, is known to be critical for leukemogenesis. To understand how the oncogenic function of IGF2BP3 impacts metabolism, we performed metabolic profiling and observed changes in glycolytic flux and one-carbon metabolism, including the biosynthesis of S-adenosyl methionine (SAM), a key substrate for methylation reactions within the cell. Using enhanced crosslinking immunoprecipitation (eCLIP) and polyribosome profiling, we found that IGF2BP3 promotes translation of the regulatory subunit of the methionine adenosyltransferase complex (MAT2B), which is involved in SAM production. Remarkably, IGF2BP3 promotes and alters the level and pattern of m^6^A modifications on mRNA. Taken together, these data suggest the intriguing hypothesis that IGF2BP3 rewrites the epitranscriptome in leukemia cells. Furthermore, this work highlights an interconnection between oncogenic metabolism and RNA modifications, suggesting that pervasive gene expression changes necessary for oncogenesis may be perpetuated by post-transcriptional gene regulation.

## In brief

Sharma et al. show that the m^6^A reader IGF2BP3 supports the production of the global methyl donor S-adenosyl methionine (SAM) by regulating one-carbon metabolism and supporting the translation of the MAT2B enzyme. MAT2B is involved in the synthesis of SAM, thus linking cancer metabolism with RNA methylation.

## Graphical Abstract

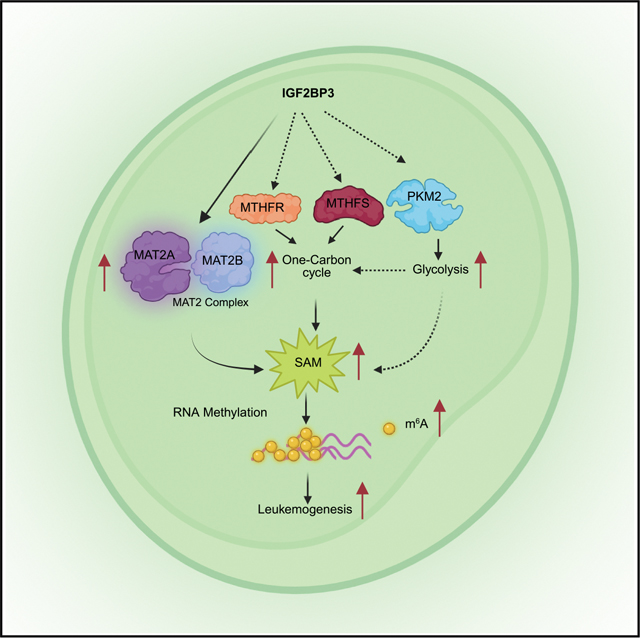

## INTRODUCTION

Methylation, the addition of a methyl group (CH_3_) to DNA, RNA, or proteins, has broad and important effects on gene expression, protein function, and cellular signaling. Although the existence of the N6-methyladenosine (m^6^A) RNA modification has been known since 1974, more recent work has revealed key regulatory roles for this RNA modification.^1–3^ The discovery of RNA methylases, followed by the identification of RNA demethylases and methylation-sensitive RNA-binding proteins (RBPs), referred to as writers, erasers, and readers, respectively, forms the basis of the epitranscriptome hypothesis, which posits that RNA modification contributes to post-transcriptional gene regulation.^4^ One of the most striking features of m^6^A methylation is its predominant localization within 3′ UTRs near the stop codon.^5,6^ This localization of m^6^A modifications, overlapping with RBP and microRNA binding sites, may underlie its reported function in a host of RNA homeostatic processes.^7^

Insulin-like growth factor 2 mRNA-binding protein 3 (IGF2BP3) is an oncofetal RBP that regulates mRNA localization, stability, and translation.^8^ The recent discovery that IGF2BP3 is an m^6^A reader is consistent with its binding preference for 3′ UTRs.^9^ IGF2BP3 regulates mRNA targets enriched for genes important in various aspects of oncogenesis and differentiation.^10–13^ Prior work from our group and others established IGF2BP3 as a critical regulator of leukemogenesis in *MLL*-translocated B-acute lymphoblastic leukemia.^11,12^ Recently, IGF2BP3 has also been shown to regulate lipid and other metabolic pathways in epithelial cancer cells.^14,15^ In this rapidly progressing field, IGF2BP3 is believed to regulate the stability of various coding RNAs, which subsequently impact enzymes that regulate different metabolic pathways.

RNA methylation is dependent on the presence of S-adenosyl methionine (SAM), which is the key methyl donor for most cellular methylation reactions. The one-carbon metabolism (OCM) pathway plays a crucial role in generating methyl donors required for DNA synthesis and DNA/RNA methylation reactions.^16^ Dysregulation of OCM has been implicated in various cancers, including leukemia, and has emerged as an essential regulator of leukemic stem cell (LSC) function.^17–19^ Serine and glycine, two key OCM metabolites, are known to play an important role in oncogenesis.^20,21^ In T cell acute lymphoblastic leukemia, serine hydroxy methyltransferases 1 and 2 (SHMT1/2) were discovered to be valuable drug targets.^22^ Together, these findings point toward an intriguing, yet not fully understood, interaction between OCM and the availability of substrates for cellular methylation reactions in cancer cells.

In our efforts to understand the effects of IGF2BP3 on leukemia cell metabolism, we uncovered an impact on glycolysis and OCM. Metabolic profiling analyses revealed a deficit in the glycolytic metabolites pyruvate and lactate, as well as those linked to OCM, including serine, glycine, SAM, and cystathionine. Importantly, this led to a reduction in total m^6^A modification, as well as in the pattern of m^6^A modification in mRNA. By adopting a combined high-throughput analysis approach using RNA-binding data from enhanced crosslinking immunoprecipitation (eCLIP) with IGF2BP3 and the metabolomics data, we identified several direct targets of IGF2BP3 in the serine-glycine cycle and the methyl/folate cycle. Western blot analysis revealed that IGF2BP3-deficient cells exhibited reduced levels of several metabolic regulators, including MAT2A and MAT2B, the rate-limiting enzymes in the production of SAM. Critically, we found that exogenous re-expression of IGF2BP3 rescued the metabolic RNA methylation phenotype. Therefore, our work reveals the unexpected phenomenon of how the m^6^A reader IGF2BP3 modulates SAM production and drives changes in RNA methylation on its target mRNAs, potentially enhancing a shift in gene expression supporting the cancerous phenotype.

## RESULTS

### IGF2BP3 promotes glycolysis in leukemic cells

**Figure 1.**
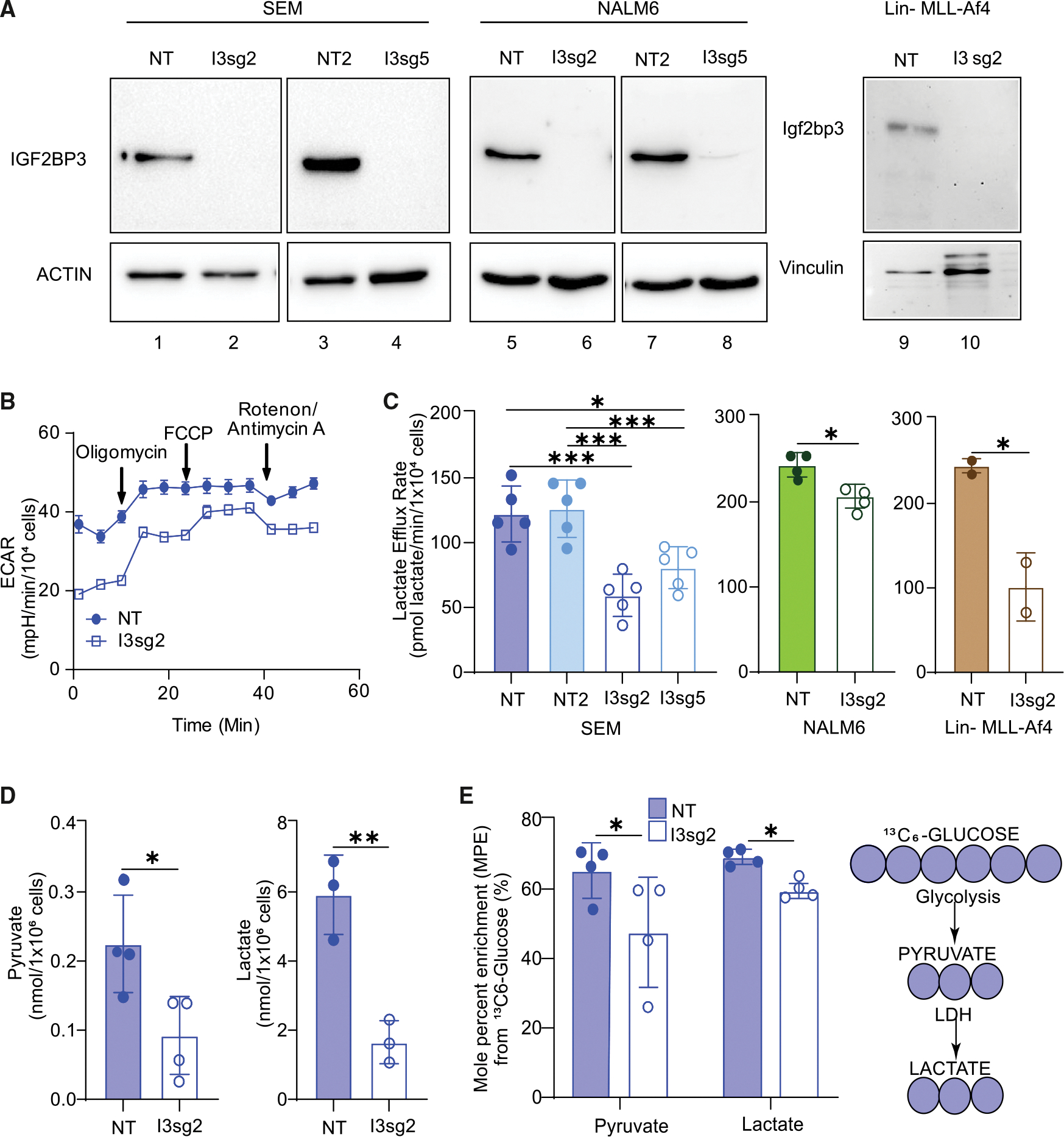
IGF2BP3 impacts glycolytic metabolism in B-acute lymphoblastic leukemia cells (A) Western blots for IGF2BP3-depleted (I3sg2 and I3sg5) SEM, NALM6, and Lin− MLL-Af4 murine cells. (B) Seahorse XF extracellular acidification rate (ECAR) kinetic trace in control and IGF2BP3-depleted SEM cells (I3sg2). (C) Aggregate lactate efflux rates from Seahorse XF analysis in control (NT and NT2) versus IGF2BP3-depleted (I3sg2 and I3sg5) SEM, NALM6, and Lin− MLL-Af4 murine cells (*n* = 2). (D) Pyruvate and lactate amounts measured by GC/MS in control versus IGF2BP3-depleted (I3sg2) SEM cells. (E) Incorporation of carbon from ^13^C-labeled glucose into pyruvate and lactate, measured as mole percent enrichment (MPE) from GC/MS experiments. All data are *n* > 3 replicates, represented as mean ± standard deviation (SD), compared by two-sided unpaired *t* test; *p < 0.05, **p < 0.01, and ***p < 0.001. All experiments were repeated at least twice for consistency. Refer also to Figure S1.

Given prior reports of IGF2BP family proteins impacting metabolism^23^ and our observations of an important role of IGF2BP3 in acute leukemia, we performed metabolic profiling experiments to understand the role of this protein in regulating cancer cell metabolism. Seahorse XF analysis showed that the depletion of IGF2BP3 decreased cellular lactate efflux, as calculated from standard Seahorse XF parameters,^24^ using independent CRISPR-Cas9 strategies in SEM cells^13^ (Figures 1A–1C, left). Reductions in the lactate efflux rate were also seen in both NALM6 cells and murine bone marrow cells transduced with MLL-Af4 depleted of IGF2BP3 (Figure 1C, middle and right). For an orthogonal measurement of glycolysis to confirm our findings, we conducted gas chromatography/mass spectrometry (GC/MS) analysis. Consistent with previous findings, we observed a reduction in steady-state levels of pyruvate and lactate (Figure 1D), as well as reduced enrichment from uniformly labeled ^13^C_6_ glucose into these metabolites (Figure 1E). To discriminate between a specific, targeted reduction in glycolytic flux or a global decrease in cellular energy demand and metabolic rate, we conducted respirometry and stable isotope tracing to identify potential changes in oxidative phosphorylation and mitochondrial function. Importantly, we did not observe reproducible changes in any oxygen consumption rate (OCR) parameters or enrichment from uniformly labeled ^13^C_6_-glucose or ^13^C_5_-glutamine into the TCA cycle, and steady-state abundances of TCA cycle intermediates were mostly unchanged (Figure S1). Altogether, the results of our initial characterization of metabolism in Figure 1 indicate that IGF2BP3 uniquely supports glycolytic flux without an appreciable effect on oxidative phosphorylation.

### IGF2BP3 supports OCM and the generation of SAM

**Figure 2.**
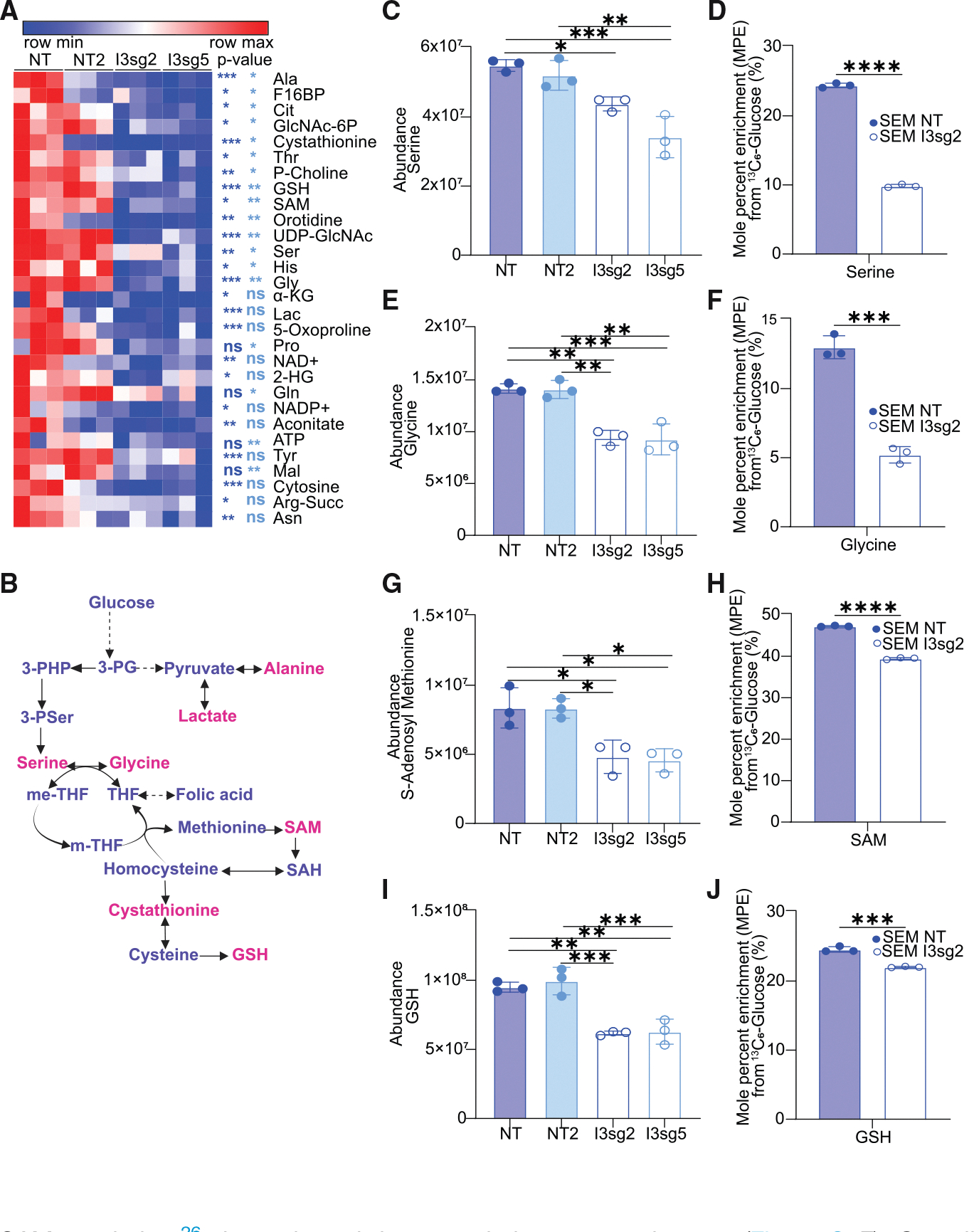
IGF2BP3 supports one-carbon metabolism pathways that serve as methyl donors (A) Heatmap depicting significantly altered metabolites from control versus IGF2BP3-depleted SEM cells, as indicated, using targeted analysis of polar central carbon metabolites by LC/MS. Shown are metabolites with a consistent change in both IGF2BP3-depleted lines, I3sg2 (purple) and I3sg5 (sky blue), with their respective *p* value significance; *n* = 3. (B) Schematic of metabolites that are produced in one-carbon metabolism. Metabolites reduced after IGF2BP3 depletion are marked in pink. (C–J) Intracellular abundance and steady-state incorporation of carbon from ^13^C-labeled glucose, measured as mole percent enrichment (MPE), into one-carbon pathway metabolites serine, glycine, S-adenosyl-methionine (SAM), and glutathione (GSH) in control versus IGF2BP3-deleted SEM cells. All data are *n* = 3 replicates represented as mean ± standard deviation (SD), compared by two-sided unpaired *t* test; *p < 0.05, **p < 0.01, and ***p < 0.001. All experiments were repeated at least twice for consistency. Refer also to Figure S2.

Given our initial findings of alterations in glycolytic metabolism in IGF2BP3-depleted cells, we undertook a liquid chromatography/MS (LC/MS) analysis of metabolites in SEM cells to more thoroughly characterize changes in glycolysis-linked pathways beyond the information available from Seahorse XF and GC/MS analysis. These LC/MS studies revealed 29 metabolites of central carbon metabolism that showed statistically significant changes in at least one of the two CRISPR knockout lines that were queried (Figure 2A) (Table S1). Looking at the metabolites noted to be altered by both GC/MS (Figure 1D) and LC/MS (Figure 2B), we found a trend toward reductions in glycolytic metabolites (lactate and fructose-1,6-bisphosphate) (Figures S2A and S2B) as well as metabolites in the one-carbon and sulfur-containing amino acid pathways, such as serine (Figure 2C), glycine (Figure 2E), glutathione (Figure 2I), and cystathionine (Figure S2C). Furthermore, isotopologue distribution patterns from uniformly labeled glucose revealed that these reduced levels were attributable to reduced synthesis (Figures 2D, 2F, 2J, and S2D, respectively). Importantly, there was a significant change in the steady-state levels of SAM, a product of the methionine cycle that derives some carbons from flux through the serine-glycine pathway (Figure 2G). The incorporation of ^13^C_6_-glucose into SAM was also reduced, again indicating that decreased flux through the serine-glycine pathway may be at least partially responsible for the reduced SAM levels (Figure 2H). Together, our findings suggest that IGF2BP3 facilitates the generation of SAM, the key methyl donor for global methylation reactions in cells.

### IGF2BP3 promotes m^6^A modifications on RNA

**Figure 3.**
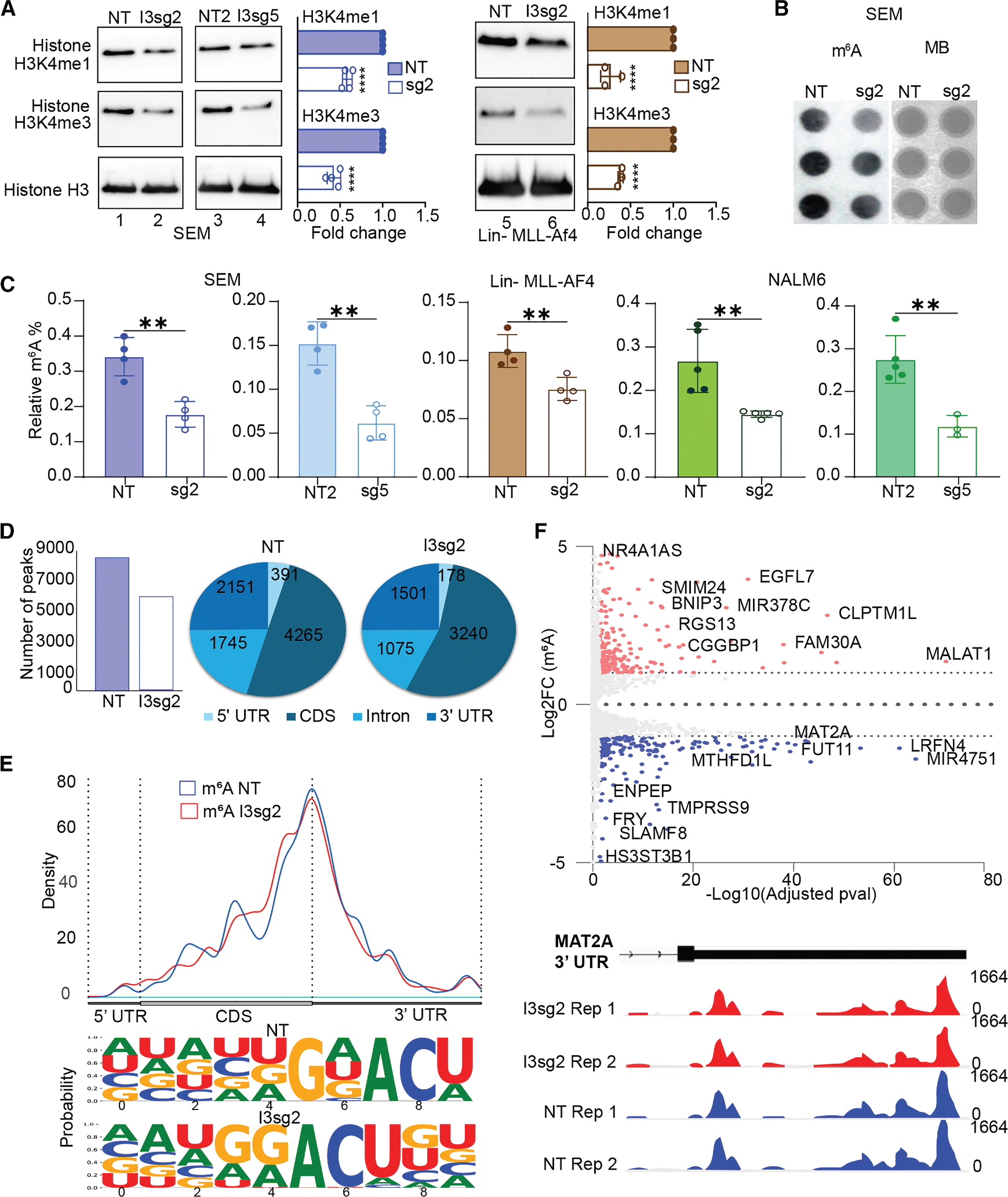
IGF2BP3 regulates N6-methyladenosine marks on RNA (A) Western blot analysis of histone methylation (H3K4me1 and H3K4me4) in SEM and Lin− MLL-Af4 cells, control or depleted for IGF2BP3; *n* = 3. (B) Dot blot analysis of m^6^A modification (left) and methylene blue staining in SEM cells, control or depleted for IGF2BP3. (C) ELISA measurement of m^6^A modification on RNA isolated from SEM, Lin− MLL-Af4, and NALM6 cells (*n* = 4 for SEM and Lin− MLL-Af4, *n* = 5 [sg5 = 3] for NALM6). (D) Bar plot (left) and pie chart (right) depicting the m^6^A peak distribution across genomic locations from the m^6^A-eCLIP data in SEM control and IGF2BP3-depleted cells. (E) Metagene plots depicting the changes in the m^6^A peak coverage across the transcriptome in SEM control and IGF2BP3-depleted cells. (F) Volcano plot (top) for genes showing differential m^6^A RNA methylation after IGF2BP3 depletion and IGF2BP3 targets defined by eCLIP analysis. Gray dashed lines indicate the significant cutoffs for differential expression (±1) and the adjusted *p* value (0.05). Hypomethylated genes are highlighted in blue, while hypermethylated genes are highlighted in red. IGV browser snapshots (bottom) of m^6^A-eCLIP depicting the coverage and change in the peak height between the NT and IGF2BP3-depleted cells for MAT2A 3′ UTR are shown. All data are *n* ≥ 3 replicates represented as mean ± standard deviation (SD), compared by two-sided unpaired *t* test; *p < 0.05, **p < 0.01, and ***p < 0.001. In case of missing or outlier values, the replicate was not reported. All experiments were repeated at least twice for consistency. All the western blots were repeated at least three times to report the changes, if any. Refer also to Figure S3.

Because SAM serves as a methyl donor for a variety of metabolic and gene regulatory processes, we hypothesized that IGF2BP3 depletion may impact protein and nucleic acid methylation. Given prior reports that SAM levels impact histone methylation,^25^ we examined H3K4me1 and H3K4me3 marks in our model systems. Indeed, both methylation marks were reduced in SEM cells (two distinct sgRNAs) and in Lin− MLL-Af4 cells that had been depleted for IGF2BP3 (Figure 3A). With this finding in hand, we next examined m^6^A marks on RNA; we performed dot blots on total RNA purified from control or IGF2BP3-depleted cells. Staining with the m^6^A antibody (see STAR Methods) was significantly reduced in IGF2BP3-depleted cells relative to control, despite equivalent levels of RNA in each sample as visualized by methylene blue staining (Figure 3B). Strikingly, IGF2BP3 deletion reduced the relative m^6^A levels in both total RNA (Figure 3C) and mRNA (Figure S3A) in both SEM and Lin− MLL-AF4 cells measured using an ELISA-based assay, confirming the reduction seen in the dot blots. Similar findings were also observed in NALM6 cells that had been depleted of IGF2BP3 (Figure 3C), where we had also noted a reduction in glycolytic flux (Figure 1C, middle). Importantly, this change in m^6^A levels was not accompanied by a concordant change in either RNA methylase or demethylase activity within the cells with the two distinct sgRNAs tested (Figures S3B and S3C). In addition, the protein levels of the canonical m^6^A writers METTL3 and METTL14, as well as METTL5, ZCCHC4, and FTO, key rRNA m^6^A writers and erasers, respectively, remained unchanged. While METTL16, another m^6^A writer involved in SAM regulation,^26^ showed a subtle upregulation, suggesting a compensatory or feedback-dependent increase in response to reduced SAM availability (Figure S3D).

To further evaluate the effect of IGF2BP3 on specific transcripts’ RNA modification status, we carried out m^6^A-eCLIP (eCLIPSE Biosciences) on poly(A)-enriched mRNA samples from control and IGF2BP3-depleted SEM cells and observed a decrease in the overall number of m^6^A peaks, with the majority of the reduction being in CDS, followed by 3′ UTRs and 5′ UTRs (Figure 3D). Some changes in the overall distribution of m^6^A modifications were apparent across the CDS (Figure 3E), as well as in the 3′UTR overlapping the IGF2BP3 binding site, as visualized from the metagene plots (Figure S3E). As anticipated, motif analysis highlighted no difference in the probability of finding the “DRACH” (D = A/G/T, R = A/G, and H = A/C/T) motif^27^ between the control and IGF2BP3-depleted cells (Figures 3E and S3E). Further, we performed differential analysis to identify significantly hypermethylated and hypomethylated regions (DMRs; number of mRNA reads positive for methylation). We found an increase in 783 m^6^A DMRs, corresponding to 223 transcripts (log2 fold change [log2FC] > 1); a decrease in 894 m^6^A DMRs, corresponding to 257 transcripts (log2FC < −1) (Figure 3F, top), one of them being MAT2A, an enzyme responsible for SAM synthesis (Figure 3F, bottom); and no change in 6,777 m^6^A DMRs, corresponding to 4,237 transcripts.

Interestingly, we also found changes in the number of m^6^A peaks on individual transcripts identified in the DMRs (Figure S3F). Overall, we identified 441 genes that exhibited a decrease in the m^6^A peak counts on their transcripts, while 1,009 genes gained m^6^A peaks on individual transcripts after IGF2BP3 depletion (Figure S3G). The metabolism-specific pathway enrichment analysis for transcripts that lost m^6^A peaks after IGF2BP3 depletion revealed pathways related to tRNA splicing (TRPT1 and TSEN54) and NAD production (QRPT1, NADSYN1, and IDO1) (Figure S3H, top), a metabolite required/generated in multiple metabolic pathways and also downregulated in the IGF2BP3-depleted cells (Figure 2A). Transcripts that gained m^6^A peak(s) after IGF2BP3 depletion revealed no significant pathways in this metabolism-focused analysis (Figure S3H, top). Similarly, Gene Ontology (GO) enrichment analysis for biological processes also revealed pathways related to the cell cycle and chromatin remodeling for transcripts losing m^6^A peaks, while no significant enrichment was found for transcripts gaining m^6^A peaks (Figure S3H, bottom) in IGF2BP3-depleted cells. While the implications of these changes require further investigation, our findings nonetheless confirm that IGF2BP3 regulates mRNA methylation.

### IGF2BP3 regulation of metabolic genes involves specific translational control

**Figure 4.**
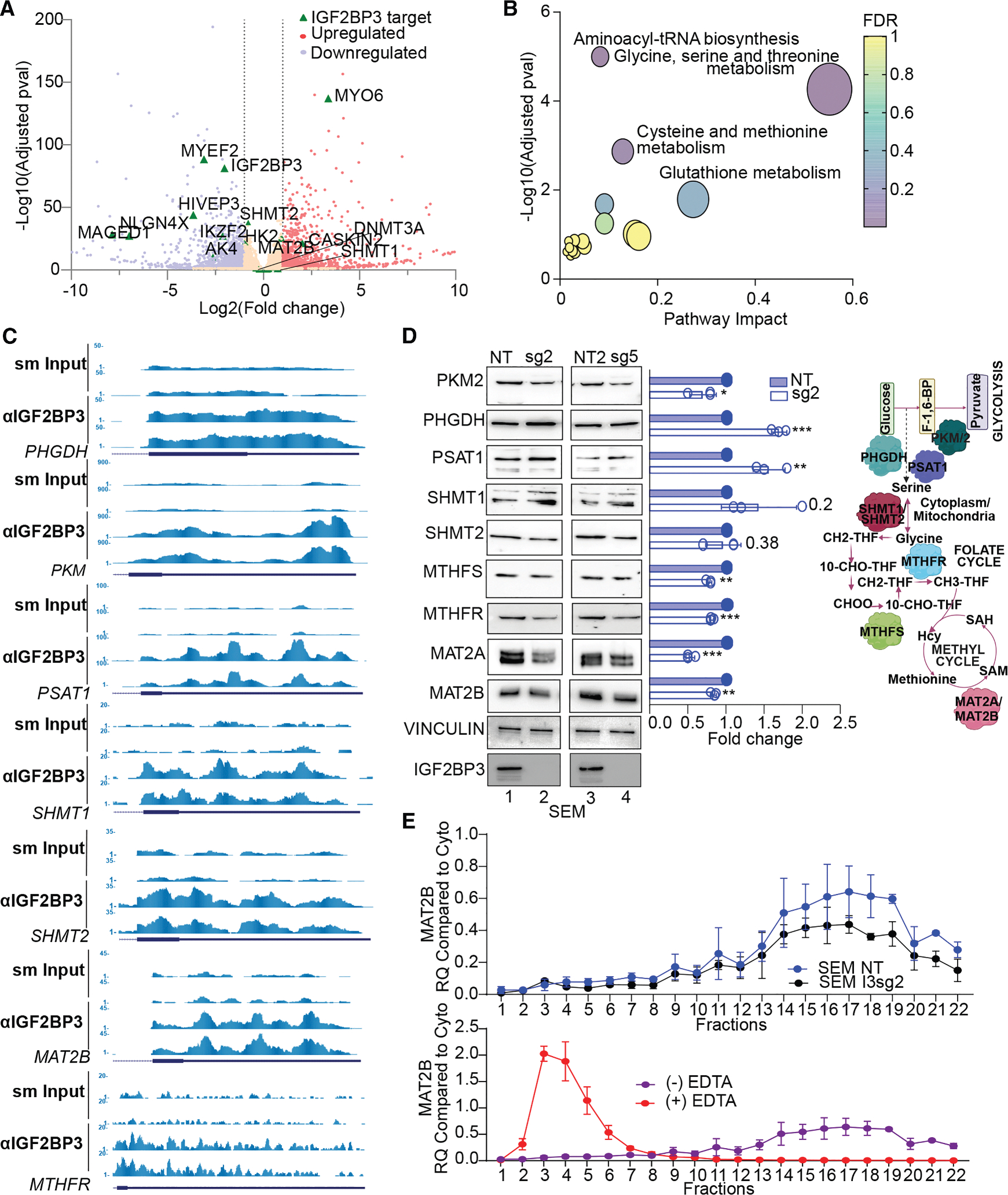
IGF2BP3 regulates translation of metabolic genes (A) Volcano plot showing differentially expressed transcripts after IGF2BP3 depletion and IGF2BP3 targets defined by eCLIP analysis (dots exceeding the thresholds depicted by dashed lines). Gray dashed lines mark the significant cutoffs for differential expression (−1/1) and the adjusted *p* value (0.05). Putative IGF2BP3 targets identified using Skipper (see Boyle et al.^29^) are highlighted as green triangles. (B) MetaboAnalyst-based pathway enrichment analysis of consistently differentially regulated metabolites after IGF2BP3 loss with IGF2BP3 eCLIP targets in SEM cells. (C) Genome browser snapshots of eCLIP read coverage across some putative IGF2BP3 target genes. Depicted are the genes with key roles in glycolysis and one-carbon metabolism, and they map to the enriched terms in (B). (D) Western blot analysis of key genes in metabolic pathways (left) and simplified schematic depiction of genes that control metabolic pathways altered in IGF2BP3-depleted cells. Numbers alongside the bars represent *p* values; *n* = 3. (E) 10%–45% sucrose gradient fractionation of cytosolic extracts from control or IGF2BP3-depleted SEM cells, along with the EDTA control. MAT2B mRNA distribution was measured by RT-qPCR and represented as mean ± standard deviation (SD) (*n* = 3). All data are *n* = 2 replicates. Data are represented as mean ± SD, compared by two-sided unpaired *t* test; *p < 0.05, **p < 0.01, and ***p < 0.001. All experiments were repeated at least twice for consistency. Refer also to Figures S4 and S5.

Our prior work^12^ demonstrated that IGF2BP3 regulates mRNA stability to drive leukemogenesis. To understand how IGF2BP3-related gene regulation may be driving the observed metabolic reprogramming, we initially performed RNA sequencing (RNA-seq) to identify differentially expressed genes in the current model. However, a majority of the genes (PKM, MAT2B, SHMT1, and SHMT2) did not exhibit changes at the RNA level, suggesting that IGF2BP3 is not regulating the stability or the steady-state mRNA levels of these metabolic regulatory genes (Figure 4A). Using an alternative analysis strategy, we used Metaboanalyst^28^ to perform a joint pathway analysis involving the integration of deregulated metabolites identified by LC/MS (Figure 2), with eCLIP data for IGF2BP3 to identify target mRNAs, in SEM cells. This analysis revealed that pathways of glycine, serine, and threonine metabolism; aminoacyl tRNA biosynthesis; and cysteine and methionine metabolism were highly and significantly enriched (Figure 4B). We further confirmed this result by analyzing the expression of key regulators of glycolysis and OCM, also identified as IGF2BP3 targets, by western blot analysis (Figure 4C). We found that there were small but consistent changes in the expression of proteins related to glycolysis, serine/glycine biosynthesis, the one-carbon cycle, and the methyl cycle (Figure 4D). Of these, the change in MAT2A (a non-target of IGF2BP3) was most concordant with a functional role in the observed metabolic changes, particularly the decrease in SAM. Broadly, the same trend in change of protein expression was also observed in the Lin− murine system (Figure S4A).

Given the surprising observation that IGF2BP3 binds to mRNAs involved in metabolic control and alters their steady-state protein levels, we explored the possibility of IGF2BP3-dependent translational control. Importantly, global translation was not altered per a SUnSET assay,^30^ where puromycin incorporation into nascent peptide chains is assessed (Figure S4B). We then profiled the polyribosome association of mRNA transcripts from control or IGF2BP3-depleted SEM cytosolic extracts using sucrose gradient centrifugation and RT-qPCR. Out of the 7 candidate transcripts identified from the eCLIP analyses (Figure 4C), we found that polyribosome association on MAT2B mRNA, which encodes the regulatory subunit of the MAT2 complex,^31^ showed a reduction in IGF2BP3-depleted cells (Figure 4E, top). Western blot analysis also revealed a decrease in steady-state protein levels in the IGF2BP3-depleted cells (Figure 4D). A similar result was obtained for the PKM gene, whereas PSAT1, SHMT1, and MTHFR transcript distribution was not significantly altered across gradients from control or IGF2BP3-depleted cells (Figures S4C–S4F, left). We confirmed that ribosome integrity was required for the observed changes, as lysates treated with EDTA, which causes ribosomal subunit dissociation, attenuate transcript sedimentation (Figures 4E, bottom, S4G, and S4C–S4G, right). Interestingly, MAT2A, the catalytic subunit of the MAT2 complex, did not exhibit any changes in polyribosome association despite showing an alteration in protein levels (Figures S5A and S5B). Given this lack of change in translation, we next queried protein stability, using a cycloheximide chase assay,^32^ and found that protein levels of MAT2A showed declining levels in IGF2BP3-deficient cells, but this was not so in IGF2BP3-expressing cells (Figure S5C). These findings are consistent with reduced stability of the MAT2A enzyme in the absence of the MAT2B subunit, as previously reported.^31^ Taken together, these results suggest that the altered translation of MAT2B, resulting from the loss of IGF2BP3, drives changes in MAT2A protein stability, in part contributing to a concomitant decrease in SAM and m^6^A in IGF2BP3-depleted cells.

### Chemical inhibition of IGF2BP3 also promotes the metabolic regulation of RNA methylation

**Figure 5.**
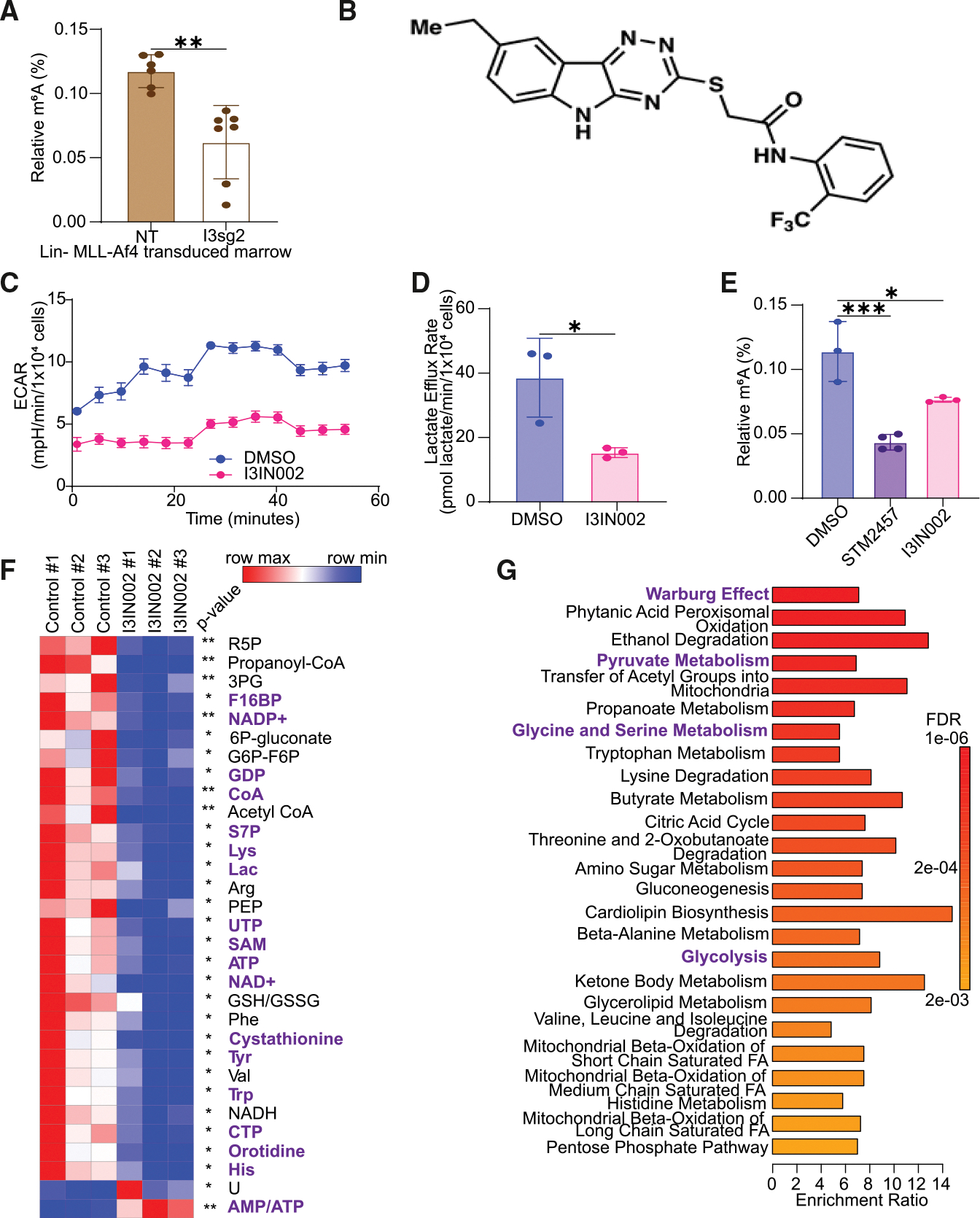
IGF2BP3 loss of function impacts glycolytic metabolism and m^6^A RNA modifications *in vivo* (A) ELISA measurement of m^6^A modification from murine bone marrow isolated following transplantation with Lin− MLL-Af4 bone marrow (see Lin et al.^13^) (*n* = 6, NT; *n* = 7, I3sg2; one-way ANOVA with Bonferroni’s test; *p < 0.05). (B) Chemical structure of I3IN-002. (C) Representative Seahorse XF extracellular acidification rate (ECAR) kinetic trace in SEM cells treated with vehicle or I3IN-002, a small-molecule inhibitor of IGF2BP3 (*n* = 4). (D) Aggregate lactate efflux rates from Seahorse XF analysis in SEM cells treated with the vehicle of I3IN-002 (*n* = 3). (E) ELISA measurement of m^6^A RNA modifications in SEM cells treated with vehicle, STM2457, and I3IN-002; *n* = 3. (F) Heatmap depicting significantly altered metabolites from DMSO-treated versus I3IN-002-treated SEM cells by LC/MS. The common metabolites that are downregulated both after IGF2BP3 deletion and after treatment with I3IN-002 are marked in purple; *n* = 3. (G) MetaboAnalyst-based pathway enrichment analysis of differentially regulated metabolites in the DMSO-treated versus I3IN-002-treated SEM cells. The common pathways that change after IGF2BP3 deletion and after treatment with I3IN-002 are marked in purple. Unless otherwise noted, all data are represented as mean ± standard deviation (SD), compared by two-sided unpaired *t* test; *p < 0.05, **p < 0.01, and ***p < 0.001. All experiments were repeated at least twice for consistency. Refer also to Figure S6.

To further investigate the significance of our observations linking IGF2BP3 to metabolism and the epitranscriptome, we sought to determine if the changes we observed were also observed after loss of function in orthogonal systems. First, we examined mouse bone marrow from mice that had been transplanted with Lin-MLL-Af4 cells, as previously described,^13^ and found that m^6^A levels were reduced with the depletion of IGF2BP3 from bone marrow cells *in vivo* (Figure 5A). Next, we turned to our recently developed small-molecule inhibitor of IGF2BP3-mRNA interactions, I3IN-002, that inhibits leukemic cell growth with an IC50 range of 1–10 μM in multiple models tested^33^ (Figure 5B). I3IN-002 inhibited cell growth with a similar IC50 in NT and METTL3-depleted SEM cells, suggesting that its inhibitory effect is independent of m^6^A RNA modifications (Figure S6A). Similar to genetic depletion, I3IN-002 reduced extracellular acidification rate (ECAR) and lactate efflux levels in SEM cells treated with 5 μM I3IN-002 (Figures 5C and 5D). I3IN-002 treatment also resulted in a reduction of m^6^A levels, similar to treatment with STM2457, a METTL3 inhibitor described recently^34^ (Figure 5E). LC/MS metabolomics studies revealed statistically significant changes in glycolytic metabolites (lactate and fructose-1,6-bisphosphate) (Figure 5F), amino acids, and one-carbon metabolites such as SAM and cystathionine (Figure 5F), similar to those observed with IGF2BP3 genetic depletion. As anticipated, metabolite enrichment analysis revealed similar pathway enrichment to that observed after IGF2BP3 depletion (Figure 5G). Immunoprecipitation of IGF2BP3 after treatment with I3IN-002 also showed significantly reduced binding to its target mRNAs, as well as a reduction at the protein level as assessed by RT-qPCR and western blot analysis, respectively (Figures S6B–S6D). Together, these data confirm, using an orthogonal chemical inhibition approach, that IGF2BP3 regulates metabolism and RNA m^6^A modifications.

### Re-expression of IGF2BP3 in knockout cells rescues glycolysis and RNA methylation

If IGF2BP3 depletion causes a reduction in glycolysis and RNA methylation, then we expect exogenous IGF2BP3 to rescue these phenotypes *in vitro*. To test this hypothesis, we re-introduced IGF2BP3 in SEM and Lin− MLL-Af4 cells in which IGF2BP3 had been depleted using CRISPR. Here, we utilized a codon-altered IGF2BP3 that retained the same amino acid sequence but had an altered nucleotide sequence to escape CRISPR-Cas9-mediated degradation (Figure S7A). Western blotting confirmed that the codon-altered protein was efficiently expressed in both model systems (Figures S7B and S7E). Re-expression of IGF2BP3 led to the rescue of cell growth and m^6^A modification of RNA in both Lin− MLL-Af4 and SEM cells (Figures S7C, S7D, S7F, and S7G, respectively). In the Lin− MLL-Af4 model, Igf2bp3 re-expression led to re-expression of MAT2A/MAT2B and PKM, while a trend toward increased expression in comparison to the knockout cells was noted in the SEM model (Figures S7B and S7E, respectively).

**Figure 6.**
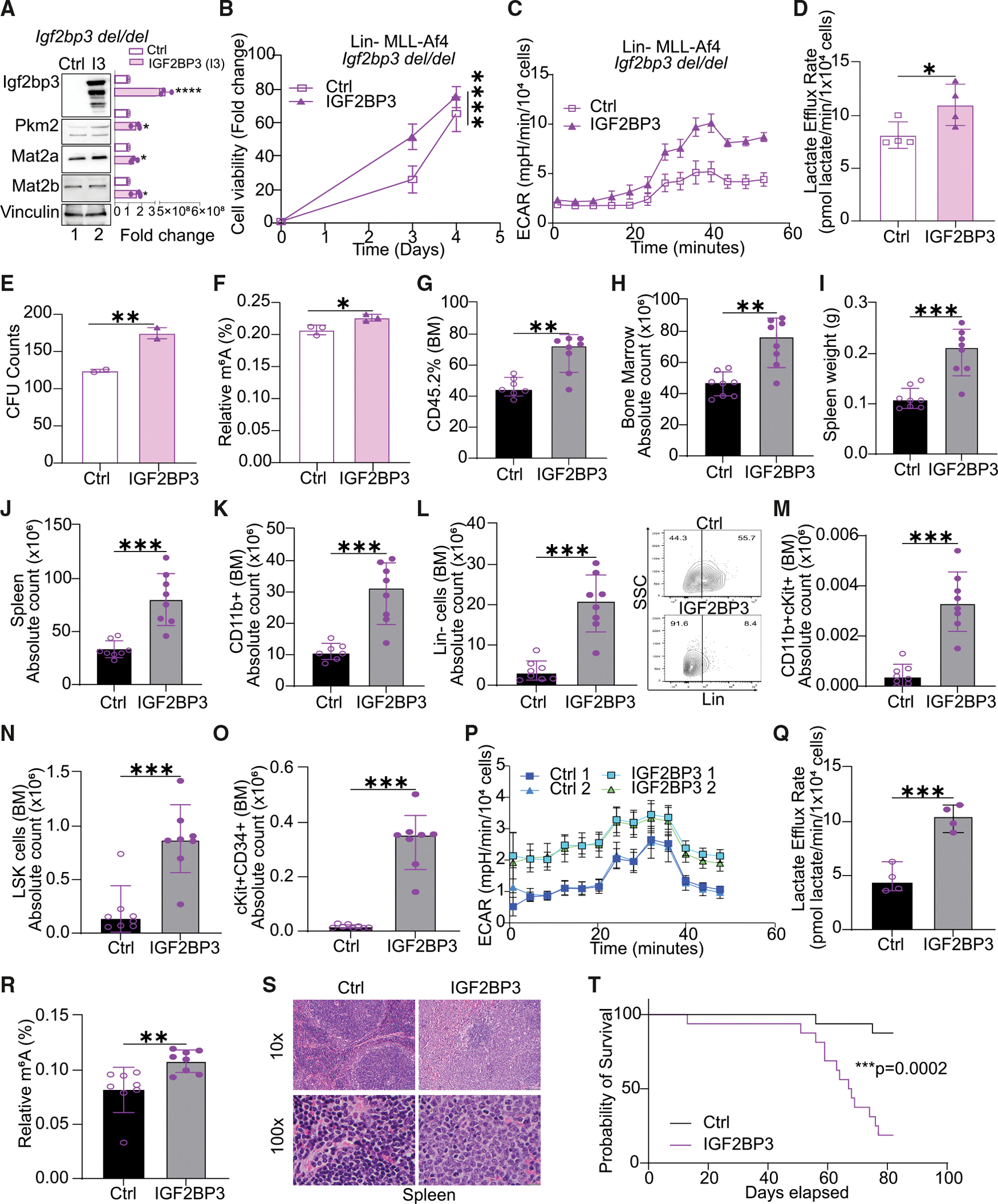
IGF2BP3 promotes glycolytic metabolism and m^6^A RNA modifications *in vivo* (A) Western blot analysis of Lin− cells from *Igf2bp3*^*del/del*^ mice. Briefly, cells were isolated from mice with a germline deletion of Igf2bp3, transformed with MLL-Af4, and then subjected to transduction with MSCV-based constructs carrying the wild-type murine Igf2bp3. Proteins that were analyzed are Igf2bp3, Mat2a, Mat2b, and actin. (B) Cell growth, measured by CellTiter-Glo, over 4 days in *Igf2bp3*^*del/del*^ Lin− MLL-Af4 cells with enforced IGF2BP3 expression as above. Viability has been normalized to control cells; mean ± standard deviation (SD) (*n* = 5); one-way ANOVA followed by Bonferroni’s multiple comparisons test; ****p < 0.0001. (C) Representative Seahorse XF extracellular acidification rate (ECAR) kinetic trace in cells described above (*n* = 4). (D) Aggregate lactate efflux rates from Seahorse XF analysis in cells described above; two-sided unpaired *t* test; *p < 0.05 (*n* = 4). (E) Colony formation assays from Lin− MLL-Af4 cells as described above; two-sided unpaired *t* test; **p < 0.01 (*n* = 2). (F) ELISA measurement of m^6^A modification on RNA isolated from *Igf2bp3*^*del/del*^ Lin− MLL-Af4 cells with enforced IGF2BP3 expression as above; two-sided unpaired *t* test; *p < 0.05 (*n* = 3). (G) Percentage engraftment of CD45.2 Lin− cells in bone marrow from *Igf2bp3*^*del/del*^ mice transduced with MLL-Af4 re-expressing empty vector (Ctrl) or IGF2BP3 in the two groups at 6 weeks. (H) Quantitation of bone marrow count in mice transplanted with MLL-Af4 re-expressing empty vector (Ctrl) or IGF2BP3 in the two groups at 6 weeks. (I) Spleen weights of mice transplanted with MLL-Af4 re-expressing empty vector (Ctrl) or IGF2BP3 in the two groups at 6 weeks. (J) Quantitation of spleen cell count in mice transplanted with MLL-Af4 re-expressing empty vector (Ctrl) or IGF2BP3 in the two groups at 6 weeks. (K) Quantitation of bone marrow CD11b+ cell count in mice transplanted with MLL-Af4 re-expressing empty vector (Ctrl) or IGF2BP3 in the two groups at 6 weeks. (L) Quantitation of bone marrow Lin− cell count along with representative fluorescence-activated cell sorting (FACS) plots in mice transplanted with MLL-Af4 re-expressing empty vector (Ctrl) or IGF2BP3 in the two groups at 6 weeks. (M) Quantitation of bone marrow CD11b+cKit+ cell count in mice transplanted with MLL-Af4 re-expressing empty vector (Ctrl) or IGF2BP3 in the two groups at 6 weeks. (N) Quantitation of bone marrow LSK (Lin− cKit+Sca1−) cell count in mice transplanted with MLL-Af4 re-expressing empty vector (Ctrl) or IGF2BP3 in the two groups at 6 weeks. (O) Quantitation of bone marrow CD11b+Sca1− (potential LIC^12^) cell count in mice transplanted with MLL-Af4 re-expressing empty vector (Ctrl) or IGF2BP3 in the two groups at 6 weeks. (P) Seahorse XF ECAR kinetic trace for bone marrow cells isolated from the empty vector (Ctrl) or IGF2BP3 re-expression group at 6 weeks (*n* = 4, each group; for representation *n* = 2). (Q) Aggregate lactate efflux rates from Seahorse XF analysis in cells described above; reported as mean ± SD (*n* = 4). (R) ELISA measurement of m^6^A RNA modifications in splenic tumors isolated from mice transplanted with MLL-Af4 re-expressing empty vector (Ctrl) or IGF2BP3 in the two groups at 6 weeks; reported as mean ± SD; 8 mice/group. (S) H&E staining of spleen of mice transplanted with mice transplanted with MLL-Af4 re-expressing empty vector (Ctrl) or IGF2BP3 in the two groups at 6 weeks. Scale bar: 100 μm. (T) Overall survival of mice transplanted with MLL-Af4 re-expressing empty vector (Ctrl) or IGF2BP3 in the two groups (representative graph cumulative of two experiments, 8 mice/group; the experiment was terminated after 12 weeks; Kaplan-Meier method with log rank test was used to report the results). The animal experiments were repeated twice. All the western blots were repeated at least three times to report the changes, if any. Data in this figure are represented as mean ± SD with *n* = 8 mice per group. Statistical tests were performed using two-sided unpaired *t* test with significance levels as indicated; *p < 0.05, **p < 0.01, and ***p < 0.001. Refer also to Figures S7 and S8.

In an orthogonal set of assays, we turned to our previously described mouse model with germline deletion of *Igf2bp3* (*Igf2bp3*^*del/del*^).^12^ Lin− cells were collected from the bone marrow of these mice and transduced with MLL-Af4 as previously described, which, in wild-type mice, leads to the overexpression of MLL-Af4 protein and efficient leukemogenesis. Next, we used retroviral transduction to constitutively express wild-type IGF2BP3 in these cells and compared it with an empty-vector control (Figure 6A). Constitutive exogenous expression of IGF2BP3 led to increased expression levels of MAT2A and MAT2B (Figure 6A), consistent with the model of gene expression regulation presented previously. Re-expression of IGF2BP3 also led to increased cell growth, as measured by cell viability in CellTiter-Glo measurements (Figure 6B). In terms of metabolic changes, we observed an increased ECAR (Figure 6C) and an increased lactate efflux rate (Figure 6D). Colony formation in methylcellulose was increased with re-expression of IGF2BP3, and importantly, m^6^A modifications in RNA were also increased (Figures 6E and 6F).

To validate the findings *in vivo*, we utilized syngeneic transplant assays to evaluate the phenotype of MLL-Af4 *Igf2bp3* (*Igf2bp3*^*del/del*^) Lin− cells with and without enforced IGF2BP3 expression. Following transplantation of transduced cells, IGF2BP3 re-expressing mice showed a significant increase in engrafted cells, bone marrow counts, spleen weights, and spleen counts at 6 weeks post-transplant compared to control mice (Figures 6G–6J). IGF2BP3 re-expressing mice displayed significantly higher counts for CD11b+, lineage− cells, and LSK (Lin− ckit+Sca1−) cells, including a potential leukemia-initiating cell (LIC) population,^12,13^ in both bone marrow and spleen (Figures 6K–6O and S8A–S8I, respectively). Next, we queried the metabolic state of cells isolated from IGF2BP3 re-expressing and control mice using respirometry. Consistent with our other findings, IGF2BP3 re-expression increased the ECAR and lactate efflux rate (Figures 6P–6Q). Importantly, we also observed an increase in the m^6^A modifications on RNA in the IGF2BP3 re-expression group compared to the control group (Figure 6R). IGF2BP3 re-expression also led to increased infiltration of the leukemic cells in the spleen and liver (Figures 6S and S8J) compared with the control mice. This was accompanied by a decreased overall survival (Figure 6T). Together, these findings confirm that IGF2BP3 upregulates leukemogenesis and RNA methylation, as well as induces metabolic changes upon re-expression.

## DISCUSSION

Despite having a 0.1%–0.4%^35^ occupancy on RNA, aberrant m^6^A RNA modification has been implicated in leukemogenesis.^36^ To date, the role of m^6^A in oncogenesis has been best elucidated in the case of m^6^A writers (METTL3/14 complex) and erasers (FTO/ALKBH5).^37–41^ This suggests that interpretation of the m^6^A modification within the cell is key to determining the effect on cellular homeostasis; in line with this idea, the YTHD family of m^6^A readers was found to play a role in oncogenesis.^42,43^ With the identification of the IGF2BP family of proteins and others as m^6^A readers, a general trend for several of these reader proteins playing critical roles in cancer pathogenesis is emerging.^9,44,45^ In this study, we report an unexpected finding that the m^6^A reader IGF2BP3 drives changes in RNA methylation via the regulation of cancer cell metabolism, potentiating post-transcriptional amplification of oncogenic gene expression.

Using a combined approach involving Seahorse XF analysis and MS, we observed significant and consistent reductions in glycolytic flux and SAM, the primary methyl donor for global methylation reactions,^46,47^ in IGF2BP3-depleted cells. Interestingly, there was no consistent decrease in oxidative phosphorylation or relative incorporation from glucose or glutamine into TCA cycle intermediates upon IGF2BP3 loss, suggesting a specific and targeted effect of IGF2BP3 on glycolysis and OCM rather than global depression of overall metabolic rates in these leukemia models. It may be that subtle changes in transcription and translation of mitochondrial proteins may not manifest in functional changes in these cells but could do so in other model systems that rely more heavily on oxidative phosphorylation to fuel energetics. Nonetheless, we did observe a specific decrease in α-ketoglutarate (α-kg) levels without commensurate changes in other TCA cycle intermediates. Given that the intracellular α-KG/succinate and α-KG/fumarate ratios regulate the α-kg-dependent dioxygenase family of demethylases and prolyl hydroxylases,^48^ it may be that IGF2BP3 regulates broader epigenetic, epitranscriptomic, and transcriptional control via modulation of these metabolites.

Given the observed impact on OCM and SAM, we queried the impact on methylation reactions within cells depleted for IGF2BP3. We observed reduced m^6^A levels following IGF2BP3 depletion. While this study was in progress, another group reported the modulation of m^6^A RNA methylation by IGF2BP3 via the m^6^A eraser FTO via overexpression of IGF2BP3.^49^ While some portions of the study are in agreement with our observations, we did not observe changes in either FTO levels or a consistent change in RNA demethylase activity with the two sgRNAs tested. In IGF2BP3-knockout cells, m^6^A-eCLIP further demonstrated a reduction in the overall m^6^A peak number but also an increase in the number of transcripts containing m^6^A peaks. The latter finding may reflect a loss of specificity in which transcripts retain m^6^A in knockout cells, with the non-canonical reader IGF2BP3 no longer stabilizing specific transcripts. Still, we observed that transcripts regulating the OCM pathway (MAT2A and MTHFD1L) exhibited decreased m^6^A peaks in IGF2BP3-depleted cells, while other transcripts showed increased m^6^A peaks. It is interesting to speculate that transcript abundance or *trans*-acting factors, such as other m^6^A readers, may play a role in determining the observed increase or decrease in transcript m^6^A content. Alternatively, some of the changes may be intrinsic to the methodology of the m^6^A-eCLIP assay.

Importantly, we found that MAT2A, a component of the MAT2 rate-limiting enzyme for SAM production, was significantly and consistently reduced. Interestingly, MAT2B mRNA, encoding the other component of the MAT2 enzyme and an allosteric regulator of MAT2A,^31^ demonstrated IGF2BP3 CLIP sites within its 3′ UTR with concordant reduction at the protein level and alteration in the polysome fractions after IGF2BP3 depletion. Prior work suggests that MAT2B binds and stabilizes functional MAT2A,^50,51^ which could explain the larger effect on MAT2A levels. Consistent with this model, MAT2A synthesis appeared to be stabilized in the presence of IGF2BP3 in a cycloheximide chase experiment (Figure S5).^31^ Together, these data support the idea that IGF2BP3 exerts translational control over the expression of key metabolic proteins to cause a shift in metabolism favoring a cellular state where methyl groups are available for RNA modifications.

There may be other factors contributing to the observed downregulation of SAM as well. Decreased glycolytic flux may play a role, including downregulation of PKM2, an isoform of *PKM* specifically overexpressed in cancers, in the absence of IGF2BP3. Interestingly, PKM2 activity is reduced in response to serine deprivation, and when in excess, serine binds to and activates PKM2 to increase glycolysis and decrease flux to serine production.^52^ A recent study also reported the involvement of NADP+, which exhibited a significant downregulation in IGF2BP3-depleted cells, in the regulation of MAT2A.^53^ We also observed downregulation in the protein levels of different genes involved in the folate cycle, which has also been linked to RNA methylation.^54,55^ Notably, some of the enzymes involved in serine and glycine biosynthesis (PHGDH, PSAT1, and SHMT1) showed upregulation in IGF2BP3-depleted cells. This may be the result of a feedback mechanism in response to decreased substrate availability.

While this manuscript was in preparation, two studies reported the regulation of serine/one-carbon metabolic pathways by IGF2BP3 in hepatocellular carcinoma.^56,57^ These studies identified different targets of IGF2BP3, which may reflect cell-type- or other context-dependent regulation. While several recent studies have demonstrated that cancer-specific gene regulatory effects of IGF2BP3 are m^6^A dependent,^58,59^ our work brings up the intriguing possibility that the effect on metabolism-related genes is m^6^A independent. Further complexity in understanding gene function arises from the fact that the basis of IGF2BP3 binding and regulation of metabolism-related mRNA is likely combinatorial based, not only on m^6^A but also sequence, spacing, and other RNA modifications, such as m^7^G, and competition with other epitranscriptome readers.^60,61^

Consistently, IGF2BP3 re-/overexpression not only rescued cell growth and leukemogenesis *in vitro* and *in vivo* but also increased m^6^A modifications in the *Igf2bp3*^*del/del*^, SEM, and Lin− MLL-Af4 systems. We also confirmed decreased m^6^A levels, as a consequence of *Igf2bp3* deletion, from *in vivo* leukemia samples.^13^ Together, these results provide multiple orthogonal lines of evidence to show that the expression of IGF2BP3 results oncogenic metabolism and changes in RNA modifications.

In conclusion, IGF2BP3, a non-canonical m^6^A reader, regulates the abundance of the m^6^A modification *in vitro* and *in vivo* via an effect on glycolysis and OCM, a critical metabolic pathway that significantly impacts cell survival and proliferation, particularly in cancer cells.^62^ These pathways involve a series of biochemical reactions that fuel the high proliferative and survival demands of cancer cells by supporting nucleotide synthesis and methylation reactions and maintaining redox balance. Our data suggest that the production of SAM via the translational control of MAT2B mRNA, and potentially others, is an important regulatory interaction controlled by IGF2BP3. Our findings provide novel insight into how m^6^A modifications may be propagated and retained in response to changes in the cellular metabolic milieu. In the future, a detailed understanding of the various aspects of IGF2BP3 function may aid in designing rational, combinatorial therapies that can pre-empt resistance and relapse.

### Limitations of the study

While our study provides evidence for the role of IGF2BP3 in the metabolic control of the cancer cell epitranscriptome, many questions remain unanswered. How do multiple RNA modifications interact to regulate IGF2BP3 function and, more broadly, other epitranscriptomic readers? How does the abundance of methyl donors impact DNA and histone methylation, and how does that impact gene expression in the context of IGF2BP3-driven oncogenesis? What is the transcript-level impact of m^6^A depletion on global translation? What accounts for the differential pattern of m^6^A gain or loss? Is the effect of IGF2BP3 on metabolism independent of m^6^A? We acknowledge that our work raises many important and interesting questions, which form several focal points for further research.

## RESOURCE AVAILABILITY

### Lead contact

Requests for further information and resources should be directed to the lead contact, Dinesh S. Rao (drao@mednet.ucla.edu).

### Materials availability

All unique/stable reagents generated in this study will be made available from the lead contact with a completed materials transfer agreement, in accordance with any additional university of funding agency policies.

### Data and code availability

- The raw data for RNA-seq, IGF2BP3 eCLIP, and m^6^A eCLIPs generated for this study have been deposited with links to BioProject accession number BioProject: PRJNA1191523 in the NCBI BioProject database (https://www.ncbi.nlm.nih.gov/bioproject/).
- Raw data of LC/MS metabolomics for the knockout and inhibitor treatment samples generated in this study are made available at the NIH Common Fund’s National Metabolomics Data Repository (NMDR) website, the Metabolomics Workbench (https://www.metabolomicsworkbench.org), where they have been assigned the project IDs PR002225 and PR002475, respectively. The data can be accessed directly via the project DOIs https://doi.org/10.21228/M81833 and https://doi.org/10.21228/M8QK08, respectively.
- Processed data from the LC/MS metabolomics experiment are available in Table S1.
- Any additional information required to reanalyze the data reported in this paper is available from the lead contact upon request.

## STAR★METHODS

### EXPERIMENTAL MODEL AND STUDY PARTICIPANT DETAILS

#### Animal studies

For *in vivo* studies, 6–8 weeks old female C57BL/6J, B6.SJL-*Ptprc*^*a*^ *Pepc*^*b,*^/BoyJ (B6 CD45.1; *RRID: IMSR_JAX:002014*), (*n* = 16) and B6J.129(Cg)-Gt(ROSA)26Sor^tm1.1(CAG-cas9^*^,−EGFP)Fezh/J^ (Cas9-GFP, BL/6J; *RRID:IMSR_JAX:026179*) transgenic mice (*n* = 2) were procured from The Jackson Laboratory. The *Igf2bp3* (*Igf2bp3*^*del/del*)^ mice used in this study were generated and maintained as previously described.^12^ All animal experiments were conducted in accordance with protocols approved by the UCLA Animal Research Committee (ARC) (Protocol number #2010–101) and were compliant with institutional guidelines and the NIH Guide for the Care and Use of Laboratory Animals. Mice were housed in the UCLA Division of Laboratory Animal Medicine (DLAM) facilities under specific pathogen–free (SPF) conditions with controlled temperature and humidity, a 12-h light/dark cycle, and *ad libitum* access to food and water. All procedures, including monitoring, anesthesia, and euthanasia, were performed to minimize pain and distress, as per ARC-approved protocols.

#### Cell lines and cell culture

All cell lines were maintained in standard conditions in an incubator at 37°C and 5% CO_2_. Human cell line HEK 293T (*ATCC CRL3216*; *RRID:CVCL_0063*) and B-ALL cell lines, NALM6 (*ATCC CRL-3273*; *RRID:CVCL_4V57*), and SEM (*DMZ-ACC 546*; *RRID:CVCL_0095*) were cultured as previously described.^75^ All cell lines used in this study were obtained from ATCC/DSMZ, which authenticates lines by short tandem repeat (STR) DNA profiling prior to distribution.

The primary murine (Lin−) cells were propagated from the bone marrow isolated either from the B6J.129(Cg)-Gt(ROSA) 26Sor^tm1.1(CAG-cas9^*^,−EGFP)Fezh/J^ (Cas9-GFP, BL/6J; *RRID:IMSR_JAX:026179*) female transgenic mice procured from The Jackson Laboratory, or the germline *Igf2bp3 (Igf2bp3*^*del/del*^*)* knockout female mice (*n* = 2) established in the lab.^12^ The Lin-cells were immortalized with MLL-Af4 by four rounds of transduction with MLL-Af4 retroviral supernatant, and selection in G418 supplemented media at 400 μg/mL for 7 days, as previously described.^12^ Cells were then stably transduced with lentiviral supernatant containing sgRNA against *Igf2bp3* (I3sg2) or non-targeting (NT), and sorted on GFP and mCherry positivity.^13,75^ For rescue experiments, germline *Igf2bp3* (*Igf2bp3*^*del/del*^) MLL-Af4 Lin-cells underwent second transduction with retroviral supernatant containing MSCV-IRES-GFP (MIG) or MSCV-IRES-GFP-IGF2BP3 (MIG-IGF2BP3) and were sorted according to GFP positivity. Immortalized MLL-Af4 transformed hematopoietic stem and progenitor cells derived from mouse bone marrow (MLL-Af4 Lin− cells) were cultured in IMDM with 15% FBS, supplemented with recombinant mouse stem cell factor (SCF, 100 ng/mL, Thermo Fisher), recombinant mouse Interleukin-6 (IL-6, 4 ng/mL, Thermo Fisher), recombinant human FMS like tyrosine kinase 3 ligand (FLT3-L, 50 ng/mL, Thermo Fisher) and mouse thrombopoietin (TPO,50 ng/mL, Thermo Fisher).

All lines were routinely tested for mycoplasma contamination using in-house PCR-based assay and found to be negative prior to experimentation.

### METHOD DETAILS

#### CRISPR/Cas9-mediated deletion/overexpression in cell lines

Human B-ALL cell lines SEM and NALM6 were depleted for IGF2BP3, while SEM for METTL3, using lentiviral delivery of CRISPR/Cas9 components in a two-vector system and sgRNA sequence as previously described^13,75^ (I3sg2; sgRNA targeted against exon 1) and I3sg5; sgRNA targeted against exon 5 of human IGF2BP3 along with two non-targeting control sgRNAs (NT and NT2). For METTL3, an sgRNA targeting exon 4 was designed. For the rescue experiments, the codon-altered IGF2BP3 (CA) sequence was synthesized and cloned into MSCV-IRES-GFP (MIG) by GeneScript (*RRID:SCR_002891*). The constructs were delivered using pGAG/POL (*RRID:Addgene_14887*) helper plasmids and pseudotyped with pVsVG (*RRID:Addgene_164440*).

The MSCV-MLL-Af4-FLAG plasmid was generously provided by Michael Thirman (University of Chicago) through a material transfer agreement.^76^ Immortalized MLL-Af4 Lin-cells were initially isolated from bone marrow of Cas9-GFP mice (*RRID:IMSR_JAX:026179*) and then transformed using retroviral transduction with MLL-Af4 retroviral supernatant, with four rounds of transduction with MLL-Af4 retroviral supernatant, followed by selection in G418 supplemented media at 400 μg/mL for 7 days, as previously described.^12^ Cells were then stably transduced with lentiviral supernatant containing sgRNA against *Igf2bp3* (I3sg2) or a non-targeting sequence (NT), and sorted based on GFP and mCherry positivity.^13,75^ For rescue experiments, germline *Igf2bp3*^*del/del*^ Lin-cells were isolated and immortalized as described above, and underwent second transduction with retroviral supernatant containing MSCV-IRES-GFP (MIG) or MSCV-IRES-GFP-IGF2BP3 (MIG-IGF2BP3), followed by sorting according to GFP positivity.

#### Protein extraction and western blot

Cell lysates were made in non-denaturing cell lysis buffer and RIPA lysis buffer and quantified using the Pierce BCA kit. Equal concentrations of lysates were electrophoresed using SDS-PAGE using standard conditions^11^ and transferred onto nitrocellulose membranes. The membranes were blocked with 5% non-fat milk, followed by sequential incubation with primary and secondary antibodies, and detected with the Pierce ECL Western Blotting Substrate (Thermo Fisher Scientific). The bands were quantified using ImageJ software^63^ (*RRID:SCR_003070*). The complete list of antibodies used is provided in the key resource table.

#### Methylcellulose-based colony-forming unit assays

The assay was performed by seeding *Igf2bp3* germline knockout and IGF2BP3 re-expressed lines into MethoCult colony-forming media (STEMCELL Technologies, M3434) at a seeding density of 500 cells/mL. Cells were cultured in MethoCult media for 10 to 12 days and then counted to determine the total number of colonies.

#### Cell proliferation, drug cytotoxicity and viability assays

*IGF2BP3-depleted* (Knockout) and NT (Non-Targeting Control) cells were seeded at 1500 cells/well in 96-well plates and cultured for 72 h at 5% CO_2_ and 37°C. CellTiter Glo (CTG) reagents were added according to the manufacturer’s instructions (Promega CellTiter-Glo kit), and luminescence was measured using a Varioskan LUX multimode microplate reader (ThermoFisher). Five technical replicates were prepared for each sample. For inhibitor treatment, cells were treated with the drug (I3IN-002 (Lab synthesized; Manuscript under consideration)) or a 0.1% dimethyl sulfoxide (DMSO) control, using concentrations and periods specified in the figure legends.

#### Cycloheximide chase assay

Briefly, 50 mg/mL cycloheximide (CHX) (01810, Sigma-Aldrich) was added to SEM cells with or without IGF2BP3 knockout at different time points, ranging from 30 min to 8 h before collection. Cells were harvested and collected for western blot analysis.

#### SUnSET assay

The assay was performed as previously described.^30^ Briefly, 50 mg/mL Puromycin (P4512, Sigma-Aldrich) was added to SEM cells with or without IGF2BP3 knockout at different time points, ranging from 45 min to 3 h before collection. Cells were harvested and collected for Western blot analysis using a monoclonal antibody against puromycin (clone 12D10 MABE343, Sigma-Aldrich) to monitor translation directly.

#### Colorimetric measurement of m^6^A levels (m^6^A ELISA)

Total RNA and PolyA-enriched mRNA (using the NEXTFLEX Poly(A) Beads Kit 2.0) were extracted from cells with or without *IGF2BP3* knockout or total RNA isolated from SEM cells treated with STM2457 (Catalog No. S9870, Selleckchem), were used to perform m^6^A ELISA. Briefly, 200 ng RNA from each condition were used, and the corresponding reagents were added according to the manufacturer’s instructions for the m^6^A detection kit (EpiGentek, USA). Finally, changes in the OD value in each well were detected by an enzymatic labeling system at a wavelength of 450 nm within 2–15 min of reagent addition. The following formula was used:

$${\text{m}}^{6}\text{A}\%\;=\;[(\text{Sample}\;\text{OD}\;-\;\text{NC}\;\text{OD})\;+\;\text{S}]\;/\;[(\text{PC}\;\text{OD}\;-\;\text{NC}\;\text{OD})\;+\;\text{P}]\;*\;100\%$$

S: The total amount of sample RNA added (ng).

P: Total amount of positive control RNA added (ng).

NC: Negative Control.

PC: Positive Control.

#### Colorimetric measurement of methylase and demethylase activity

RNA methylase and demethylase activity were measured using the commercially available Epigenase m^6^A Methylase Activity/Inhibition Assay Kit (Epigentek; P-9019) and Demethylase Activity/Inhibition Assay Kit (Epigentek; P-9013), respectively. The assays were performed per the manufacturer’s protocol. Briefly, 10 μg of total protein lysate was used for control and knockouts. The samples were incubated on the plate for 90 min, washed with wash buffer, and incubated with capture antibody, detection antibody, and enhancer antibody for 60, 30, and 30 min, respectively. After washing five times with a wash buffer, the developer solution was added, and the color change was monitored for 5 min. The reaction was stopped using a stop solution, and the absorbance was read at 450 nm using a Varioskan Lux multimode microplate reader (Thermo Fisher). Methylase and Demethylase activity were reported in units of OD/h/mg and normalized against the standard curve.

#### Respirometry

All oxygen consumption and extracellular acidification measurements were conducted using an Agilent Seahorse XF Pro or XF^e^96 Analyzer. Experiments were performed at 37°C and pH 7.4. All respiratory parameters were corrected for non-mitochondrial respiration and background signal from the instrument with the addition of 200 nM rotenone and 1 μM antimycin A. Where appropriate, oligomycin was used at 2 μM unless otherwise specified, and FCCP concentrations were titrated to determine the optimal concentration for a given experiment. Unbuffered DMEM assay medium was composed of DMEM (Sigma #5030; pH 7.4) supplemented with 31.6 mM NaCl, 3 mg/L phenol red, and 5 mM HEPES unless otherwise indicated.

#### GC/MS analysis and stable isotope tracing

Experiments were performed as described previously.^77^ Metabolite extraction was performed using a Folch-like method with a 5:2:5 ratio of methanol: water: chloroform. For the isotope tracing experiment, 5 million SEM cells per technical replicate were plated in a medium containing either 10 mM ^13^C_6_ glucose (Cambridge Isotope Laboratories) or 6 mM ^13^C_5_ glutamine (Cambridge Isotope Laboratories) for 24 h. After incubation, the cells were washed with ice-cold 0.9% (w/v) NaCl and then resuspended in a mix of ice-cold methanol, water containing 5 μg/mL norvaline (Sigma #N7502; an internal standard) and chloroform. Samples were then vortexed for 1 min and centrifuged at 10,000g for 5 min at 4°C. The polar fraction (top layer) was removed, and the samples were dried overnight using a refrigerated CentriVap vacuum concentrator (LabConco). Metabolites (50 nmol–23 pmol) were extracted alongside the cell samples to ensure the signal fell within the linear detection range of the instrument. The dried polar metabolites were reconstituted in 20 μL of 2% (w/v) methoxyamine in pyridine prior to a 45-min incubation at 37°C. Subsequently, 20 μL of MTBSTFA with 1% tertbutyldimethylchlorosilane was added to samples, followed by an additional 45-min incubation at 37°C. Samples were analyzed using Agilent MassHunter software (*RRID:SCR_015040*) and FluxFix software (http://fluxfix.science; *RRID:SCR_026259*) to correct for the abundance of natural heavy isotopes against an in-house reference set of unlabeled metabolite standards.^64^

#### Metabolite extraction and mass-spectrometry-based metabolomics analysis

SEM cells with (WT/Control: NT, NT2), or without IGF2BP3 (knockout (KO; sg2, sg5)) or SEM cells treated with (Ctrl: DMSO), or IGF2BP3 inhibitor (I3IN-002) were cultured in their regular culture medium without glucose but supplemented with ^13^C_6_ Glucose (10 mM, Cambridge Isotope Laboratories, Inc.). 24 h later, 5×10^6^ cells were collected and rinsed with PBS, and 1 mL cold 80% methanol (Optima* LC/MS, Fisher Scientific) was added to cells. As an internal standard, 1 μM norvaline (Sigma-Aldrich) was added to each sample. Samples were then vortexed every 5 min, three times and spun down at top speed for 5 min at 4°C. The supernatants were transferred to a newtubes, and the pellet was resuspended in 0.5 M NaOH for protein estimation. The extracts were dried overnight using a refrigerated CentriVap vacuum concentrator (LabConco) and stored at −80°C. The mass spectrometry-based analysis of extracted metabolites was conducted at UCLA Metabolomics Center. Dried metabolites were resuspended in 100 μL 50% ACN:water and 5 μL was loaded onto a Luna 3μm NH2 100A (150 × 2.0 mm) column (Phenomenex). The chromatographic separation was performed on a Vanquish Flex (Thermo Scientific) with mobile phases A (5 mM NH4AcO pH 9.9) and B (ACN) and a flow rate of 200 μL/min. A linear gradient from 15% A to 95% A over 18 min was followed by 7 min isocratic flow at 95% A and re-equilibration to 15% A. Metabolites were detected with a Thermo Scientific Q Exactive mass spectrometer run with polarity switching in full scan mode with an m/z range of 70–975 and 70.000 resolution. Maven (v 8.1.27.11; *RRID:SCR_022491*) was utilized to quantify the targeted metabolites by AreaTop using accurate mass measurements (<5 ppm) and expected retention time previously verified with standards. Values were normalized to cell number or protein content of extracted material, where applicable. ^13^C natural abundance corrections were made using AccuCor^65^ (*RRID:SCR_023046*). Total amounts were calculated by summing up the intensities of all detected isotopologues of a given metabolite. Data analysis was performed using in-house R scripts. MetaboAnalyst (v6.0; *RRID:SCR_015539*) was then used to analyze the enriched metabolic pathways of significantly changed metabolites with default parameters.^28^ Heatmap was generated using Morpheus (https://software.broadinstitute.org/morpheus; *RRID:SCR_014975*) and reported only those that showed statistically significant changes in at least one of the two CRISPR knockout lines that were queried, with the most significant (significant in both replicates) at the top. To determine the carbon contribution from tracer glucose in each metabolite, the Mole percent enrichment (MPE) was calculated as a percentage by dividing the sum of ^13^C labeled enriched carbons by the total number of ^12^C carbons.

#### Sucrose gradient preparation for polysome profiling

Linear sucrose gradients (15–45%) were made by dissolving sucrose in a polysome gradient buffer (20 mM Tris-HCl, pH 7.5, 200 mM KCl, 25 mM MgCl_2_). Approximately 6.5 mL of the 15% sucrose solution was layered into SW41 centrifuge tubes, followed by the 45% solution underneath. The gradients were mixed using the Biocomp Gradient Station, then stabilized at 4°C overnight.

#### Polyribosome profiling and fractionation

The experiment was performed as previously described.^78^ Briefly, SEM cells were treated with 100 μg/mL CHX for 10 min at 37°C, then pelleted (200 rpm for 10 min). The pellet was resuspended in 9 mL PBS (without MgCl_2_ and KCl) and pelleted again. After removing the supernatant, cells were lysed in an ice-cold lysis buffer (20 mM Tris-HCl pH 7.5, 200 mM KCl, 25 mM MgCl_2_, 0.5% NP-40, 100 μg/mL Cycloheximide, supplemented with protease inhibitors). For the EDTA treatment of polyribosomes, the polysomes were washed with the polysome buffer (50 mM EDTA, 25 mM MgCl_2_, supplemented with protease inhibitors and without Cycloheximide). The lysate was passed through a 23-gauge needle 3–5 times and incubated on ice for 10 min. Following incubation, the lysate was centrifuged (10,000 rcf for 10 min at 4°C) to separate nuclear and membrane fractions. The cytosolic supernatant was then layered onto the pre-formed sucrose gradient and ultracentrifuged at 40,000 rpm for 2 h at 4°C using a Beckman SW41 rotor. The gradients were manually fractionated (non-continuous) from top to bottom into 22 samples of 0.4 mL each using the Biocomp Gradient Station (Piston Gradient Fractionator (Model 153)). Fractions were pooled into 96-well plates with a 1:1 ratio of 2x RNA Shield containing spike-in RNA (SARS-CoV N1).

#### RNA extraction and cDNA synthesis for polysome profiling

Total RNA was extracted from cell culture pellets using an Agilent Bravo automated liquid handling platform and Quick-DNA/RNA Viral Magbead kit following the manufacturer’s protocol (Zymo Research). Samples were treated with RQ1 RNase-Free DNase following the manufacturer’s protocol (Promega). cDNA synthesis was then performed using a High-Capacity cDNA Reverse Transcription Kit following the manufacturer’s protocol (Applied Biosystems). cDNA was diluted 1:10 and qPCR was performed.

#### Polysome profiling qPCR

qPCR experiments were performed on a QuantStudio 6 Flex Real-Time PCR System (Applied Biosystems). Experiments were performed using Luna Universal qPCR Master Mix, following the manufacturer’s protocol (New England Biolabs). SARS-CoV2 N1 RNA spike-in was used to normalize the relative expression levels of target mRNAs to the cytosol fraction with the ΔΔCt method. Primer sequences are provided in Table S2.

#### RNA-immunoprecipitation

RNA immunoprecipitation assay was performed on SEM cells treated with 5 μM concentration of I3IN-002 and DMSO for 48 h. After incubation, cells were lysed in a non-denaturing lysis buffer (0.5% NP40, 150 mM KCl, 1 mM NaF, 25 mM Tris, 2 mM EDTA) supplemented with protease inhibitor and RNase inhibitor. Lysates were cleared at 12000 rpm for 15 min 10% of the precleared lysate was stored as ‘Input’. The precleared lysates were incubated overnight with Protein G Agarose beads along with antibody against human IGF2BP3 (MBL, RN009P) or normal mouse IgG (sc-2025). Pelleted beads were washed thrice with wash buffer (300 mM NaCl, 50 mM Tris, 0.01% NP40, 5 mM MgCl_2_) and resuspended in Non-Denaturing Buffer the next day. The “Input” and the re-suspended beads were equally distributed and dissolved in Laemmli SDS buffer for Western blot and in TRIzol (Thermo Scientific) for RNA isolation and qRT-PCR.

#### cDNA preparation and qRT-PCR analysis

RNA was isolated from the RIP samples, and then 1 μg from each sample was reverse-transcribed using qScript cDNA super mix (VWR). qRT-PCR was performed on the cDNA samples to quantify the gene expression of IGF2BP3 targets: PKM2, MAT2B, PSAT1, and SHMT2 using PerfeCTa SYBR Green Fast Mix (VWR). 18S was used as an internal control. ΔΔCt method was used to compare the gene expression.^79^ The qPCR primer sequences used are provided in Table S2.

#### Animal experiments

For *in vivo* studies, *Igf2bp3* (*Igf2bp3*^*del/del*^) mice used in this study were generated and maintained as previously described.^12^ Briefly, 8-week-old CD45.1+ recipient *C57BL/6J, B6.SJL-Ptprca Pepcb,/BoyJ (B6 CD45.1; RRID: IMSR_JAX:002014)* female mice were treated with Busulfan. A total sample size (*n*) of 16 mice (*n* = 8 Ctrl and *n* = 8 MLL-Af4 for IGF2BP3 overexpression) were used for each set of experiments. For rescue experiments, 1 × 10^6^ MSCV-IRES-GFP (Control) or MSCV-IRES-GFP-IGF2BP3 (MIG-IGF2BP3) overexpressed *Igf2bp3 (Igf2bp3*^*del/del*^*)* donor Lin-cells immortalized with MLL-Af4 were injected and allowed to be transplanted in the CD45.1+ recipients as described previously.^12^ Flow cytometry was performed to check the peripheral blood engraftment of the leukemic cells at weeks 2 and 4. Once the peripheral blood engraftment reached >20%, the experiment was terminated, and tissues were harvested to be analyzed by Flow cytometry, histology and m^6^A ELISA. Flow cytometry data was analyzed by FlowJo v10.8 Software (BD Life Sciences; *RRID:SCR_008520*). All the animal experiments received Institutional Animal Research Committee approval at UCLA and were repeated twice.

#### Histopathology

Tissues (Spleen, liver, kidney, and thymus) were fixed in formaldehyde and embedded in paraffin using standard histological protocols in the Translational Pathology Core Laboratory (TPCL), UCLA. The tissues were then sectioned and stained with Hematoxylin & Eosin dyes. Stained sections were visualized and imaged using an Olympus BX-41 light microscope. Analysis was performed by a board-certified hematopathologist (DSR).

#### eCLIP-seq

eCLIP studies were performed by Eclipse Bioinnovations Inc https://eclipsebio.com/according to the published single-end eCLIP protocol.^80^ Briefly, 20×10^6^ SEM cells were grown and UV cross-linked at 400 mJoules/cm^2^ with 254 nm radiation, flash frozen, and stored until use at −80°C. Crosslinked cell pellets were further processed by Eclipse Bioinnovations for eCLIP using a rabbit anti-IGF2BP3 antibody (MBL, RN009P) or anti-m^6^A antibody. A parallel Size-Matched Input (SMInput) library was also generated as a control, where the samples were treated identically, except they were not immunoprecipitated with anti-IGF2BP3 or anti-m^6^A antibody. Protein-RNA complexes were separated on an SDS-PAGE gel, transferred to a PVDF membrane and isolated using standard iCLIP protocol.^81^ Libraries were then amplified as previously described.^82^ Three replicates using 20 million SEM cells per replicate (3 IP libraries and 3 size-matched input libraries for IGF2BP3 and 2 IP libraries and 2 size-matched input libraries for m^6^A) were processed, yielding six and four libraries, respectively. Sequencing was performed using the SE72 protocol on the NextSeq platform.

#### eCLIP-seq read processing

Data were processed similarly to the standard eCLIP pipeline.^82^ Briefly, reads were trimmed (cutadapt; *RRID:SCR_011841*) and FASTQs were aligned with STAR v2.7.8a^66^ (*RRID:SCR_004463*) to the human genome (GRCh38, GENCODE v38 annotation). To remove all the repetitive elements, a reverse intersection of all peak files with the repeatmasker bed file (downloaded from the University of California Santa Cruz (UCSC) table viewer; *RRID:SCR_005780*) was performed.^67^ PCR duplicate reads were removed, and the aligned files were further processed and analyzed for peaks enriched over the background using Skipper v1.0.0^29^ (*RRID:SCR_026260*). IGF2BP3 and m^6^A eCLIP fine-mapped peak sets were filtered for peaks with log2 (fold change) ≥ 1.0 and ≥3.0, respectively, in terms of mean read counts in IP vs. size-matched input.^82^ Differentially methylated peaks in m^6^A I3sg2 and m^6^A NT samples were analyzed using DESeq2–1.32.0^68^ (*RRID:SCR_015687*). The count files for each replicate were subjected to differential binding analysis to identify statistically significant differences between I3sg2 and NT. Python was used for visualizing metagene, m^6^A heatmap and volcano plot (*RRID:SCR_008394*). The *p*-values from the Wald tests are displayed as the y axis of volcano plot, with the log2 fold change of normalized peak intensities (I3sg2 over NT) as the x axis. The m^6^A coverage plots for the differential m^6^A peak height in the NT and I3sg2 samples were depicted using the IGV genome browser^74^ (*RRID:SCR_011793*).

#### Motif analysis

Identification of known and *de novo* motif sequence enrichment for m^6^A in the NT and I3sg2 and IGF2BP3 in the NT samples was performed using findMotifsGenome.pl from Homer2 (*RRID:SCR_009586*) with default and modified parameter for RNA motifs.

#### m^6^A peak count per transcript analysis

The differentially methylated peaks were annotated including 5′UTR, CDS and 3′UTRs using the transcriptome-based annotations from GENCODE v38 as mentioned before. To explore the number of peak differences per transcript, a cumulative number of peak scores per transcript for respective gene was calculated from the peak annotation file and the same was visualized as a heatmap in I3sg2 and NT SEM cells. The gene enrichment analysis and visualization of the data was performed using ggplot2 package of R on RStudio.^72,73^

#### Joint analysis of metabolomics and eCLIP datasets

For finding the enriched metabolic pathways, using the IGF2BP3 eCLIP datasets, MetaboAnalyst^28^ (v6.0) “joint pathway analysis” module was used. For the enrichment analysis, a list of significantly changed metabolites along with IGF2BP3’s mRNA target list was used with default parameters.^28^ Additionally, for the enrichment of metabolic pathways using HumanCyc^70^ (*RRID: CR_002298*) and Metabolomics Workbench Metabolites^71^ (*RRID:SCR_013794*) from the IGF2BP3 knockout datasets, enrichR^69^ (*RRID:SCR_001575*) web module was used.

#### RNA extraction

Total RNA was extracted with TRI Reagent (Zymo Research) using the manufacturer’s protocol for the RNA isolation with the following modification: one additional RNA ethanol wash step was included. After the total RNA was solubilized in ddH_2_0, one overnight ethanol precipitation step was included for further purification of the total RNA.

#### Illumina sequencing of mRNA libraries

Total RNA was isolated for cell culture pellets as described above. 2 μg of total RNA for each sample was used for mRNA library preparation using the NEXT- FLEX Rapid Directional RNA-Seq Kit 2.0 following the manufacturer’s protocol (PerkinElmer Applied Genomics). Before library preparation, total RNA samples were subjected to Poly(A) selection and purification using the NEXTFLEX Poly(A) Beads Kit 2.0 following the manufacturer’s protocol (PerkinElmer Applied Genomics). Pooled mRNA sequencing libraries were sequenced on an Illumina NovaSeq S4 at the UC Davis Sequencing Core Facility, generating 150 bp paired-end reads. Normalization and differential gene expression (DGE) were done using DESeq2 (v1.32.0; *RRID:SCR_015687*).^68^

#### m^6^A dot blots

Total RNA was isolated from cell culture pellets as described above, and three concentrations, starting from 100 μg with serial dilutions, were used for blotting. RNA was first denatured for 5 min at 95° C and then placed on ice for 5 min. Hybond-N^+^ nylon membrane (Amersham Biosciences) was pre-soaked for 5 min in 2x SSC before RNA blotting was performed using a commercial apparatus with a vacuum manifold (Schleicer and Schuell, Inc.). The membrane was cross-linked twice using the auto-cross link function on a UV Stratalinker 2400. The membrane was then blocked for 1 h at room temperature in a 10% blocking solution/0.1% PBST before incubation overnight at 4°C with primary m^6^A antibody (Millipore MABE-1006) (1:1,000) in 5% blocking solution/0.1% PBST. The membrane was washed 3 times for 5 min in 0.1% PBST and incubated for 1 h at room temperature in HRP mouse secondary antibody (1:10,000) in 5% blocking solution/0.1% TBST. The membrane was washed 3 times for 5 min in 0.1% PBST and then incubated for 5 min in SuperSignal West Pico PLUS substrate (Thermo Scientific). The membrane was then visualized using a BioRad Chemidoc using the optimal auto-exposure setting. As a loading control, samples were run in parallel on a separate membrane and stained with 0.1% methylene blue for two hours before destaining in ddH_2_O until the dots were visible.

### QUANTIFICATION AND STATISTICAL ANALYSIS

The data shown represents mean ± SD for continuous numerical data. Statistical tests comparing two conditions were employed as follows: Two-tailed student’s T test for data showing a normal distribution. For multiple conditions, one-way ANOVA was used, followed by Bonferroni’s multiple comparisons test for multiple comparisons. Statistical tests for non-high-throughput data were performed using GraphPad Prism software (*RRID:SCR_002798*) and described in the figure legends. Survival analyses were performed using the Kaplan-Meier method, with comparisons made using log rank tests, followed by Bonferroni’s correction for multiple comparisons. A *p*-value less than or equal to 0.05 was considered significant. Details of the statistical tests, sample sizes, number of replicates, and definition of error bars are provided in the figure legends.

## Supplementary Material

1

2

Supplemental information can be found online at https://doi.org/10.1016/j.celrep.2025.116330.

## Figures and Tables

**KEY RESOURCES TABLE:** 

REAGENT or RESOURCE	SOURCE	IDENTIFIER

Antibodies		

PKM2	Proteintech	15822-1-AP; RRID: AB_1851537
PHGDH	Thermo Fisher	PA5-80896; RRID: AB_2788160
PSAT1	Thermo Fisher	PA5-121028; RRID: AB_2914600
SHMT1	Proteintech	30192-1-AP; RRID: AB_2935529
SHMT2	Proteintech	11099-1-AP; RRID: AB_2188452
MTHFS	Thermo Fisher	PA5-118092; RRID: AB_2902694
MTHFR	Thermo Fisher	PA5-140282; RRID: AB_2927284
MAT2A	Proteintech	55309-1-AP; RRID: AB_2881303
MAT2B	Proteintech	15952-1-AP; RRID: AB_10637268
METTL3	Proteintech	15073-1-AP; RRID: AB_2142033
METTL14	Proteintech	26158-1-AP; RRID: AB_2800447
METTL16	Proteintech	19924-1-AP; RRID: AB_10639364
FTO	Proteintech	27226-1-AP; RRID: AB_2880809
m^6^A	Sigma Aldrich	MABE-1006; RRID: AB_3674612
Histone H3	Thermo Fisher	PA516183; RRID: AB_10985434
H3K4ME3	Thermo Fisher	MA511199; RRID: AB_10977872
H3K4me1	Thermo Fisher	710795; RRID: AB_2532764
IGF2BP3	Proteintech	14642-1-AP; RRID: AB_1570642
IGF2BP3	MBL	RN009P; RRID: AB_2122782
ACTIN	Sigma Aldrich	A5441; RRID: AB_476744
VINCULIN	Santa Cruz	sc-73614; RRID: AB_2941767
Normal mouse IgG	Santa Cruz	Sc-2025; RRID: AB_737182
GAPDH	GeneTex	GTX627408; RRID: AB_11174761
METTL5	Proteintech	16791-1-AP; RRID: AB_2142051
ZCCHC4	CST	64933S; RRID: AB_3697102
ZCCHC4	Antibodies.com	A16979; RRID: AB_3697103
Puromycin	Sigma Aldrich	MABE343, clone12D10; RRID: AB_3674613
APC anti-mouse CD3ε Antibody	Biolegend	100311; RRID: AB_312676
APC/Cyanine7 anti-mouse CD117 (c-kit) Antibody	Biolegend	105826; RRID: AB_1626278
PE/Cyanine7 anti-mouse/human CD11b Antibody	Biolegend	101215; RRID: AB_312798
PerCP/Cyanine5.5 anti-mouse Ly-6A/E (Sca-1) Antibody	Biolegend	108124; RRID: AB_893615
BD Horizon^™^ BV786 Mouse Anti-Mouse CD45.2	BD Biosciences	563686; RRID: AB_2738375
Biotin anti-mouse CD4 Antibody 500 ug	Biolegend	100404; RRID: AB_312689
Biotin anti-mouse TCR γ/δ Antibody 50 ug	Biolegend	118103; RRID: AB_313827
Biotin anti-mouse/human CD11b Antibody	Biolegend	101203; RRID: AB_312786
IgM Monoclonal Antibody (II/41), Biotin, eBioscience^™^, Invitrogen^™^	Fisher	13-579-082; RRID: AB_3674614
Biotin anti-human CD45 Antibody	Biolegend	368534; RRID: AB_2721498
PE/Cyanine7 anti-human CD19 Antibody	Biolegend	302215; RRID: AB_314245
PerCP/Cyanine5.5 anti-human CD34 Antibody	Biolegend	343521; RRID: AB_1937272
Biotin anti-mouse/human CD45R/B220 Antibody, 500 ug	Biolegend	103204; RRID: AB_312989
Biotin anti-mouse Ly-6G/Ly-6C (Gr-1) Antibody, 500ug	Biolegend	108404; RRID: AB_313369
Biotin anti-mouse NK-1.1 Antibody, 500 ug	Biolegend	108704; RRID: AB_313391
Biotin anti-mouse TER-119/Erythroid Cells Antibody, 500ug	Biolegend	116204; RRID: AB_313705
Biotin anti-mouse TCR β chain Antibody, 500ug	Biolegend	109204; RRID: AB_313427
Biotin anti-mouse CD8a Antibody, 500ug	Biolegend	100704; RRID: AB_312743
PE/Cyanine7 anti-mouse CD16/32 Antibody, 100ug	Biolegend	101318; RRID: AB_2104156
eBioscience^™^ Streptavidin eFluor^™^ 450 Conjugate	LifeTech	48-4317-82; RRID: AB_10359737
CD34 Monoclonal Antibody (RAM34), Alexa Fluor 700, eBioscience^™^, 100ug	LifeTech	56-0341-82; RRID: AB_493998
BD Horizon^™^ BV605 Mouse Anti-Mouse CD45.2	BD Biosciences	563051; RRID: AB_2737974

Bacterial strains		

5-alpha Competent *E. coli*	Thermo Fisher Scientific	Cat# EC0112

Chemicals, peptides & reagents		

L-Glutamine (200mM)	Thermo Fisher Scientific	Cat# 25030-081
^13^C_6_-Glucose	Cambridge Isotope Laboratories, Inc.	Cat# CLM-1396
Halt protease inhibitor cocktail	Thermo Fisher Scientific	Cat# 78442
DPBS	Thermo Fisher Scientific	Cat# 14040117
DL-Norvaline	Sigma-Aldrich	N7502
G418	Thermo Fisher Scientific	Cat# 11811-031
Cycloheximide	Sigma-Aldrich	Cat# 01810
Recombinant Human Flt3-Ligand	Gibco	Cat# PHC9415
Recombinant mouse SCF	Gibco	Cat# 25003100UG
Recombinant mouse TPO	Gibco	Cat# 31514100UG
Recombinant mouse IL-6	Gibco	Cat# PHC0065
Puromycin dihydrochloride	Sigma-Aldrich	Cat# P4512
Penicillin Streptomycin	Thermo Fisher Scientific	Cat# 15-140-122
Halt Protease and Phosphotase inhibitors cocktail 100x	Thermo Fisher Scientific	Cat# PI-78440
SuperSignal West Pico PLUS substrate	Thermo Fisher Scientific	Cat# 34580
Streptavidin MicroBeads	Miltenyi Biotec	Cat# 130-048-101
Busulfan	Sigma-Aldrich	Cat# B2635
I3IN-002	Lab stock	Recipe available upon request
STM2457	Selleckchem	Cat# S7890
DMSO	Sigma-Aldrich	Cat# D2650
RIPA Buffer	Thermo Fisher Scientific	Cat# NC9484499
TRI Reagent	Zymo Research	Cat# R2050
TRIzol Reagent	Thermo Fisher Scientific	Cat#15596026
BioT DNA Transfection Reagent	Bioland	Cat# B01-01
PerfeCTa^®^ SYBR^®^ Green FastMix^®^, ROX^™^	VWR	Cat# 101414-278
qScript^™^ cDNA SuperMix	VWR	Cat# 101414-106
Fetal Bovine Serum, dialyzed, US origin, One Shot^™^ format	Thermo Fisher Scientific	Cat# A3382001
IMDM (Iscove’s Modified Dulbecco’s Medium)	Thermo Fisher Scientific	Cat# 12440053
DMEM, high glucose	Thermo Fisher Scientific	Cat# 11-995-073
RPMI 1640 Medium	Thermo Fisher Scientific	Cat# 11-875-093
DMEM, no glucose, no glutamine, no phenol red	Thermo Fisher Scientific	Cat# A1443001

Commercial assay kits		

Thermo Scientific^™^ Pierce^™^ BCA^™^ Protein Assay	Thermo Fisher Scientific	Cat# PI23225
MethoCult colony-forming media	STEMCELL Technologies	Cat# M3434
Quick-DNA/RNA Viral Magbead kit	Zymo Research	Cat# R2140
Luna Universal qPCR Master Mix	NEB	Cat# M3003
CellTiter-Glo(R) Luminescent Cell Viability	Promega	Cat# G7572
EpiQuick m6A RNA Methylation Quantification Kit	Epigentek	Cat# P-9005
Epigenase m6A Demethylase Activity/Inhibition Assay Kit	Epigentek	Cat# P-9013
Epigenase m6A Methylase Activity/Inhibition Assay Kit	Epigentek	Cat# P-9019
QIAGEN Plasmid Maxi Kit	QIAGEN	Cat# 12362
NEXT- FLEX Rapid Directional RNA-Seq Kit 2.0	Perkin Elmer Applied Genomics	Cat# NOVA-5198-01
NEXTFLEX Poly(A) Beads Kit 2.0	Perkin Elmer Applied Genomics	Cat# NOVA-512991

Deposit data		

RNA-seq (Raw data)	This study	BioProject: PRJNA1191523
IGF2BP3 eCLIP-seq (Raw data)	This study	BioProject: PRJNA1191523
m^6^A eCLIP-seq (Raw data)	This study	BioProject: PRJNA1191523
m^6^A IGF2BP3 KO (sg2) eCLIP-seq (Raw data)	This study	BioProject: PRJNA1191523
IGF2BP3 knockout (Metabolomics (LC/MS) Raw data)	This study	NMDR: PR002225
I3IN-002 treatment (Metabolomics (LC/MS) Raw data)	This study	NMDR: PR002475
Metabolomics (LC/MS) Analyzed data	This study	Table S1

Experimental models: Cell lines		

HEK293T	ATCC	CRL-3216; CVCL_0063
NALM6	ATCC	CRL-3273; CVCL_4V57
SEM	DSMZ	ACC 546; CVCL_0095
Lin- MLL-Af4 & derived clones	Lab stock and This study	N/A
Lin- *Igf2bp3 ^del/del^* MLL-Af4 & derived clones	This study	N/A
SEM derived clones	This study	N/A

Experimental models: Organisms/strains		

B6J.129(Cg)-Gt(ROSA)26Sor^tm1.1(CAG-cas9*,–EGFP)Fezh/J^ (Cas9-GFP, BL/6J)	The Jackson Laboratory	Strain #:026179; IMSR_JAX:026179
C57BL/6J, B6.SJL-Ptprca Pepcb,/BoyJ (B6 CD45.1)	The Jackson Laboratory	Stock No: 033076; IMSR_JAX:002014
*Igf2bp3^de/del^*	Tran et al.^12^	N/A

Recombinant DNA		

MSCV-IRES-GFP-IGF2BP3-CA	This study	Synthesized from GenScript; SCR_002891
MSCV-IRES-GFP	Palanichamy et al.^11^	N/A
MSCV-IRES-GFP-IGF2BP3	Palanichamy et al.^11^	N/A
MSCV-MLL-Af4-FLAG	Tran et al.^12^	Kind gift from Michael Thirman (University of Chicago)
pLKO5-sgIGF2BP3-1	This study	N/A
pLKO5-sgIGF2BP3-2	This study	N/A
pLKO5-sgNT	This study	N/A
pLKO5-sgNT2	This study	N/A
pLKO5-sgMETTL3-2	This study	N/A
PsPax2	(Addgene#12260)	Kind gift from Didier Trono; Addgene_12260
pMD2.G	(Addgene#12259)	Kind gift from Didier Trono; Addgene_12259
pLKO5.sgRNA.EFS.tRFP	(Addgene#57823)	Kind gift from Benjamin Ebert; Addgene_57823
pLenti-Cas9-GFP	(Addgene#86145)	Kind gift from David Sabatini; Addgene_86145
pCL-ECO	(Addgene#12371)	Kind gift from Inder Verma; Addgene_12371
pCL-GAG/POL	(Addgene#14887)	Kind gift from Tannishtha Reya; Addgene_14887
pHDM-VSV-G	(Addgene#164440)	Kind gift from Alejandro Balazs; Addgene_164440
MSCV-hU6-mCherry-EFS	Lin et al.^13^	N/A
MSCV-hU6-mCherry-EFS-sgIgf2bp3-1	Lin et al.^13^	N/A
MSCV-hU6-mCherry-EFS-sgNT	Lin et al.^13^	N/A

Oligonucleotides		

DNA primers or oligos, see Table S2	N/A	Integrated DNA Technologies (IDT)

Software & Algorithm		

ImageJ	Schneider et al.^63^	https://imagej.net/ij/; RRID: SCR_003070
Agilent MassHunter software	N/A	https://www.agilent.com/en/promotions/masshunter-mass-spec; RRID: SCR_015040
FluxFix software	Trefely et al.^64^	http://fluxfix.science; RRID: SCR_026259
Maven (v 8.1.27.11)	N/A	http://maven.princeton.edu/index.php; RRID: SCR_022491
AccuCor	Su et al.^65^	https://cran.r-project.org/web/packages/accucor/index.html; RRID: SCR_023046
MetaboAnalyst (v6.0)	Pang et al.^28^	https://www.metaboanalyst.ca/MetaboAnalyst/ModuleView.xhtml; RRID: SCR_015539
Morpheus	N/A	https://software.broadinstitute.org/morpheus/; RRID: SCR_014975
Cutadapt (version 3.4)	N/A	http://code.google.com/p/cutadapt/; RRID: RRID: SCR_011841
STAR v2.7.8a	Dobin et al.^66^	http://code.google.com/p/rna-star/; RRID: SCR_004463
UCSC Genome Browser	Karolchik et al.^67^	https://genome.ucsc.edu/; RRID: SCR_005780
Skipper	Boyle et al.^29^	https://github.com/YeoLab/skipper; RRID: SCR_026260
GraphPad Prism	N/A	https://www.graphpad.com/features; RRID: SCR_002798
DESeq2-1.32.0	Love et al.^68^	http://www.bioconductor.org/packages/release/bioc/html/DESeq2.html; RRID: SCR_015687
EnrichR	Kuleshov et al.^69^	https://maayanlab.cloud/Enrichr/; RRID: SCR_001575
BioCyc	Karp et al.^70^	https://biocyc.org/; RRID: SCR_002298
Metabolomics Workbench	Sud et al.^71^	https://www.metabolomicsworkbench.org/; RRID: SCR_013794
FlowJo v10.8	FlowJo	https://www.flowjo.com/solutions/flowjo; RRID: SCR_008520
Python3	N/A	https://www.python.org/; RRID: SCR_008394
Homer2	N/A	http://homer.ucsd.edu/homer; RRID: SCR_009586
R	R Core Team^72^	https://www.R-project.org/; RRID: SCR_001905
RStudio	Posit team^73^	http://www.posit.co/; RRID: SCR_000432
ggplot2	N/A	https://cran.r-project.org/web/packages/ggplot2/index.html; RRID: SCR_014601
Biorender	N/A	https://BioRender.com; SCR_018361
IGV Browser	Robinson et al.^74^	https://igv.org/; RRID: SCR_011793

## Highlights

- IGF2BP3 regulates one-carbon metabolism
- IGF2BP3 supports the translation of the MAT2B, a subunit of the MAT2 complex
- Enforced expression of IGF2BP3 promotes m^6^A marks on RNA both *in vitro* and *in vivo*
