# Supplementary material for: IGF2BP3 redirects glycolytic flux to promote one-carbon metabolism and RNA methylation
Source: Cell Rep. Author manuscript; Available in PMC 2025 Nov 7. (PMC12593248; doi:10.1016/j.celrep.2025.116330)
Supplement: 1 [file NIHMS2120101-supplement-1.pdf]

Cell Reports, Volume *44*

Supplemental information

IGF2BP3 redirects glycolytic flux to promote one-carbon metabolism and RNA methylation

Gunjan Sharma, Martin Gutierrez, Anthony E. Jones, Shruti Kapoor, Amit Kumar Jaiswal, Zachary T. Neeb, Amy Rios, Poornima Dorairaj, Michelle L. Thaxton, Tasha L. Lin, Tiffany M. Tran, Lyna E.S. Kabbani, Alexander J. Ritter, Georgia M. Scherer, Jacob P. Sorrentino, Linsey Stiles, Johanna ten Hoeve, Robert D. Damoiseaux, Neil K. Garg, Ajit S. Divakaruni, Jeremy R. Sanford, and Dinesh S. Rao

Figure S1

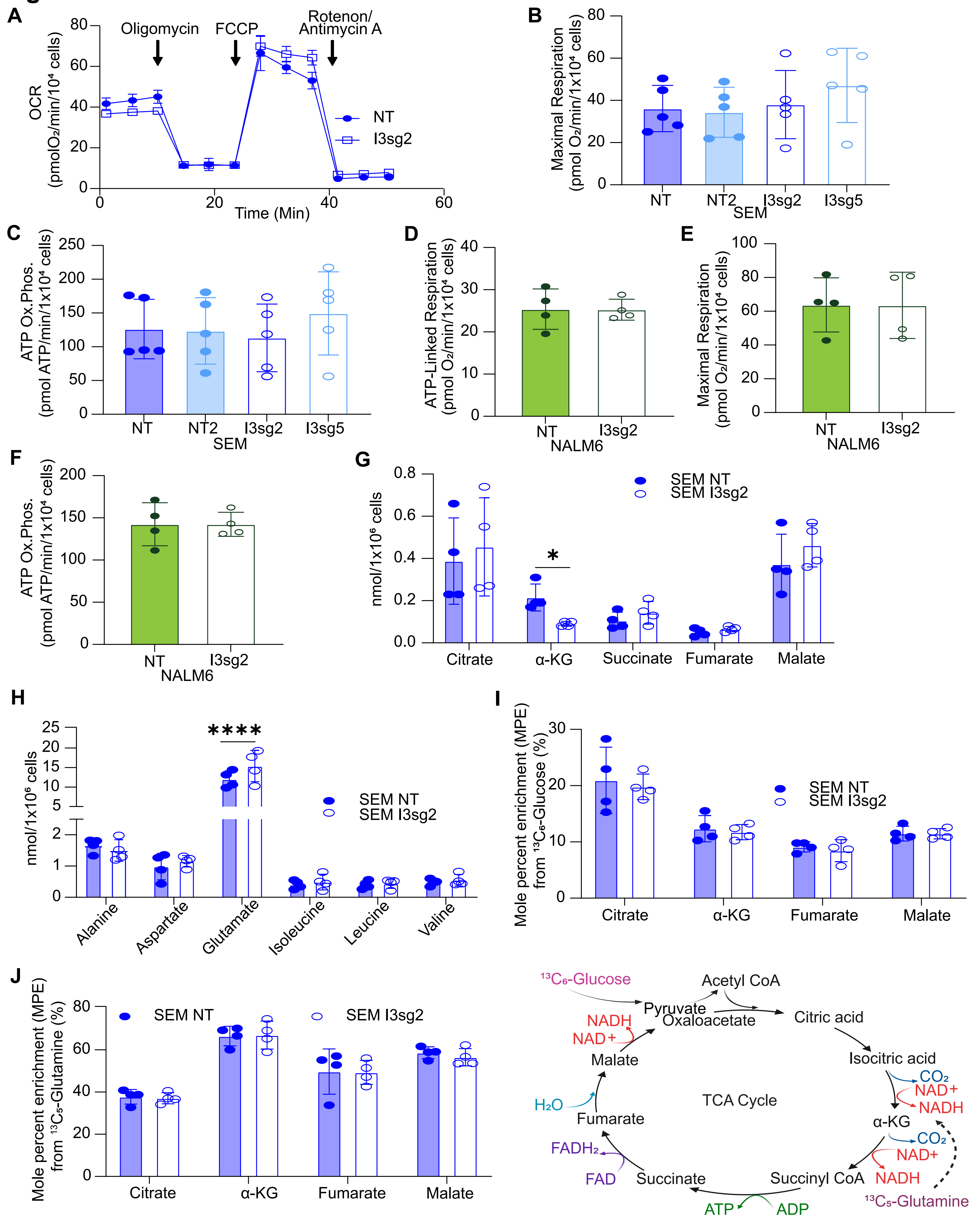

A
OCR (pmolO2/min/10⁴ cells)
Oligomycin
FCCP
Rotenon/ Antimycin A
NT
I3sg2
Time (Min)
B
Maximal Respiration (pmol O2/min/1x10⁴ cells)
NT
NT2
I3sg2
I3sg5
SEM
C
ATP Ox.Phos. (pmol ATP/min/1x10⁴ cells)
NT
NT2
I3sg2
I3sg5
SEM
D
ATP-Linked Respiration (pmol O2/min/1x10⁴ cells)
NT
I3sg2
NALM6
E
Maximal Respiration (pmol O2/min/1x10⁴ cells)
NT
I3sg2
NALM6
F
ATP Ox.Phos. (pmol ATP/min/1x10⁴ cells)
NT
I3sg2
NALM6
G
nmol/1x10⁶ cells
SEM NT
SEM I3sg2
Citrate
α-KG
Succinate
Fumarate
Malate
H
nmol/1x10⁶ cells
SEM NT
SEM I3sg2
Alanine
Aspartate
Glutamate
Isoleucine
Leucine
Valine
I
Mole percent enrichment (MPE) from ¹³C6-Glucose (%)
SEM NT
SEM I3sg2
Citrate
α-KG
Fumarate
Malate
J
Mole percent enrichment (MPE) from ¹³C5-Glutamine (%)
SEM NT
SEM I3sg2
Citrate
α-KG
Fumarate
Malate
¹³C6-Glucose
Acetyl CoA
Pyruvate
Oxaloacetate
Citric acid
NADH
NAD+
Malate
Isocitric acid
CO2
NAD+
NADH
TCA Cycle
H2O
Fumarate
α-KG
CO2
NAD+
FADH2
NADH
FAD
Succinate
Succinyl CoA
¹³C5-Glutamine
ATP
ADP

Figure S2

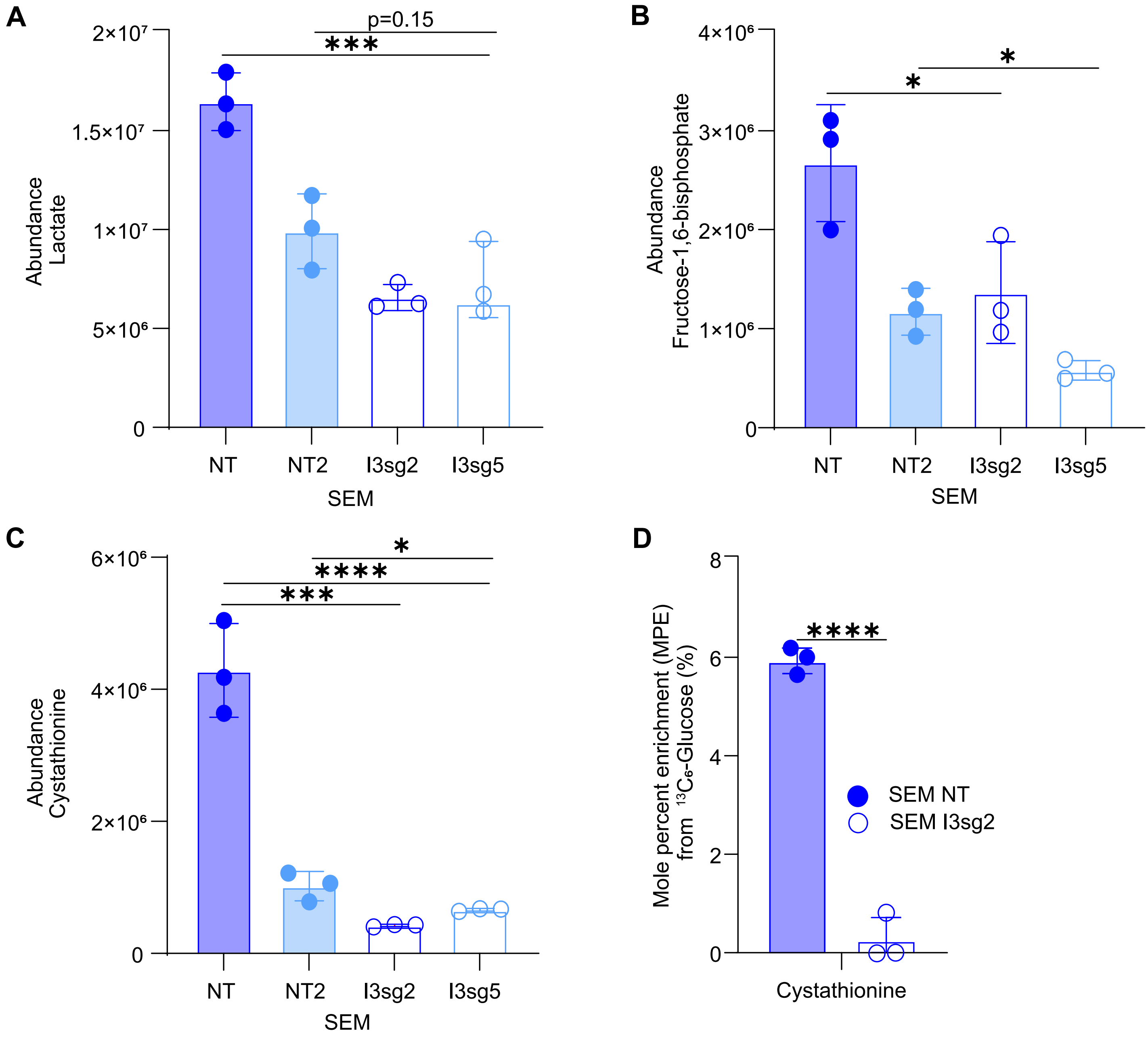
A
p=0.15
***
2×10⁷
1.5×10⁷
1×10⁷
5×10⁶
0
Abundance
Lactate
NT
NT2
I3sg2
I3sg5
SEM
B
*
*
4×10⁶
3×10⁶
2×10⁶
1×10⁶
0
Abundance
Fructose-1,6-bisphosphate
NT
NT2
I3sg2
I3sg5
SEM
C
*
****
***
6×10⁶
4×10⁶
2×10⁶
0
Abundance
Cystathionine
NT
NT2
I3sg2
I3sg5
SEM
D
****
8
6
4
2
0
Mole percent enrichment (MPE)
from ¹³C₆-Glucose (%)
SEM NT
SEM I3sg2
Cystathionine

Figure S3

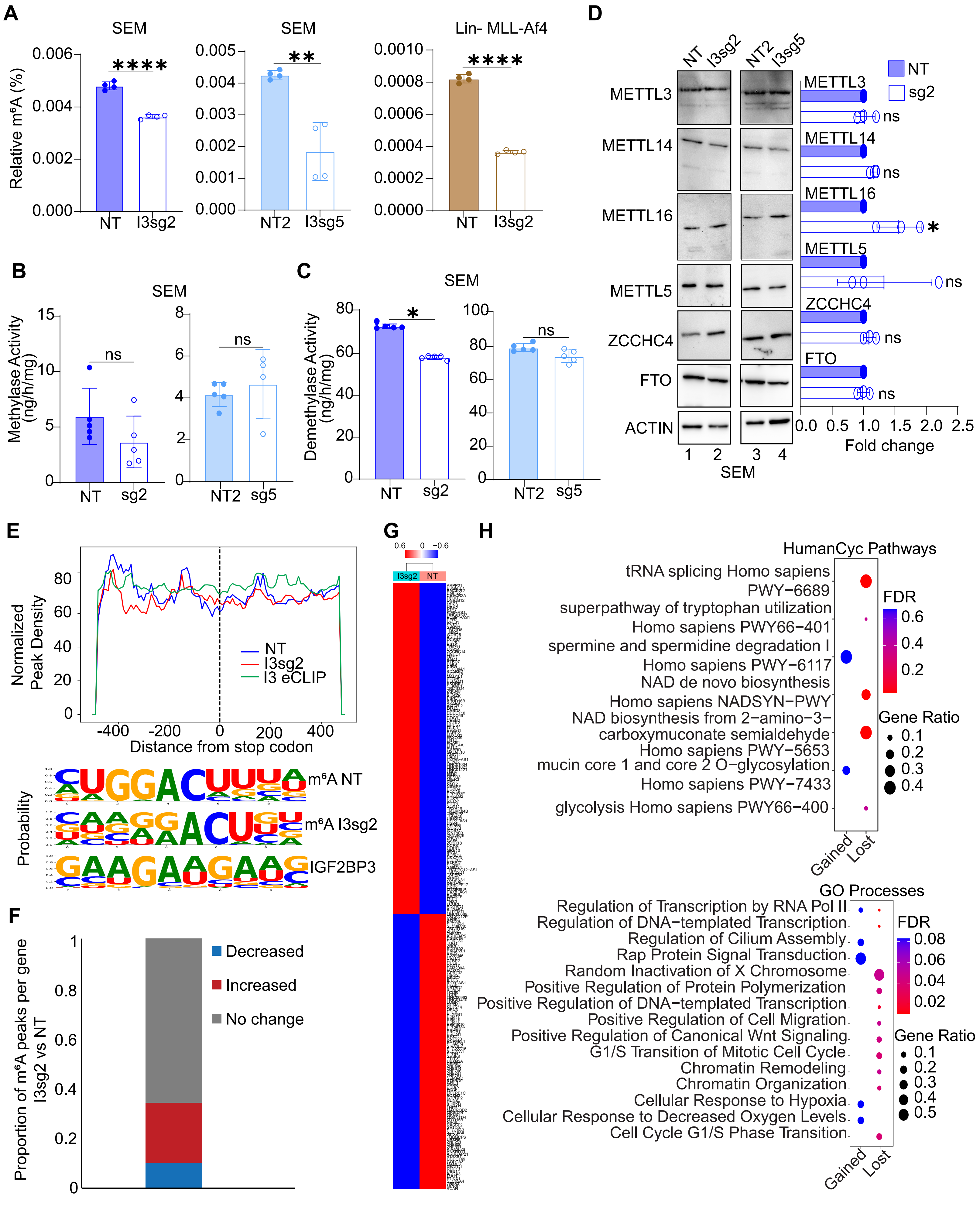

A
SEM
SEM
Lin- MLL-Af4
Relative m⁶A (%)
0.006
0.004
0.002
0.000
****
NT
I3sg2
0.005
0.004
0.003
0.002
0.001
0.000
**
NT2
I3sg5
0.0010
0.0008
0.0006
0.0004
0.0002
0.0000
****
NT
I3sg2
B
SEM
Methylase Activity (ng/h/mg)
15
10
5
0
ns
NT
sg2
8
6
4
2
0
ns
NT2
sg5
C
SEM
Demethylase Activity (ng/h/mg)
80
60
40
20
0
*
NT
sg2
100
80
60
40
20
0
ns
NT2
sg5
D
NT
I3sg2
NT2
I3sg5
METTL3
METTL14
METTL16
METTL5
ZCCHC4
FTO
ACTIN
1
2
3
4
SEM
NT
sg2
METTL3
ns
METTL14
ns
METTL16
*
METTL5
ns
ZCCHC4
ns
FTO
ns
0.0
0.5
1.0
1.5
2.0
2.5
Fold change
E
Normalized Peak Density
80
60
40
20
0
-400
-200
0
200
400
Distance from stop codon
NT
I3sg2
I3 eCLIP
Probability
m⁶A NT
m⁶A I3sg2
IGF2BP3
F
Proportion of m⁶A peaks per gene I3sg2 vs NT
1
0.8
0.6
0.4
0.2
0
Decreased
Increased
No change
G
0.6
0
−0.6
I3sg2
NT
H
HumanCyc Pathways
tRNA splicing Homo sapiens PWY−6689
superpathway of tryptophan utilization Homo sapiens PWY66−401
spermine and spermidine degradation I Homo sapiens PWY−6117
NAD de novo biosynthesis Homo sapiens NADSYN−PWY
NAD biosynthesis from 2−amino−3−carboxymuconate semialdehyde Homo sapiens PWY−5653
mucin core 1 and core 2 O−glycosylation Homo sapiens PWY−7433
glycolysis Homo sapiens PWY66−400
FDR
0.6
0.4
0.2
Gene Ratio
0.1
0.2
0.3
0.4
Gained
Lost
GO Processes
Regulation of Transcription by RNA Pol II
Regulation of DNA−templated Transcription
Regulation of Cilium Assembly
Rap Protein Signal Transduction
Random Inactivation of X Chromosome
Positive Regulation of Protein Polymerization
Positive Regulation of DNA−templated Transcription
Positive Regulation of Cell Migration
Positive Regulation of Canonical Wnt Signaling
G1/S Transition of Mitotic Cell Cycle
Chromatin Remodeling
Chromatin Organization
Cellular Response to Hypoxia
Cellular Response to Decreased Oxygen Levels
Cell Cycle G1/S Phase Transition
FDR
0.08
0.06
0.04
0.02
Gene Ratio
0.1
0.2
0.3
0.4
0.5
Gained
Lost

Figure S4

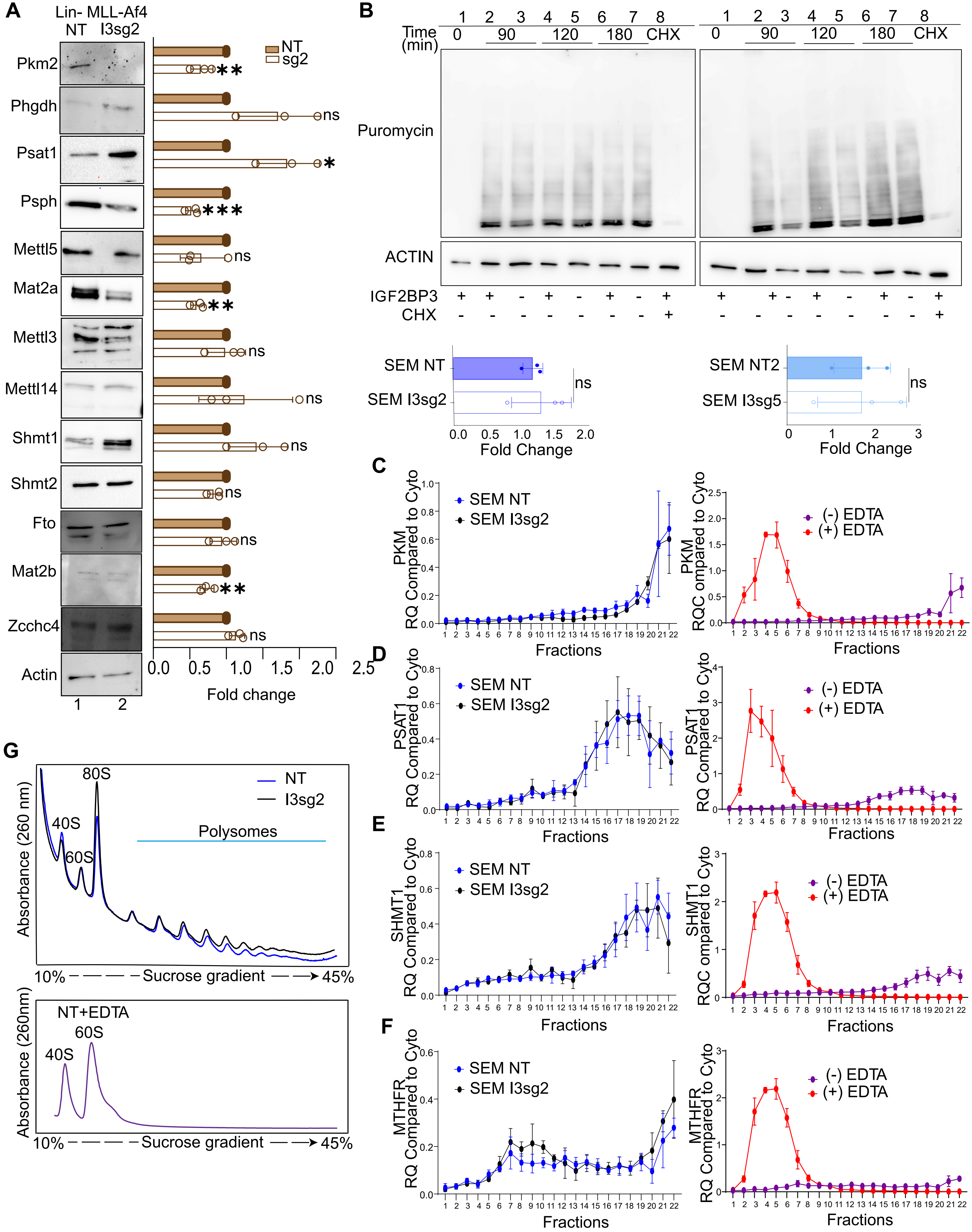
A
Lin- MLL-Af4
NT
I3sg2
Pkm2
Phgdh
Psat1
Psph
Mettl5
Mat2a
Mettl3
Mettl14
Shmt1
Shmt2
Fto
Mat2b
Zcchc4
Actin
1
2
NT
sg2
**
ns
*
***
ns
**
ns
ns
ns
ns
ns
**
ns
0.0
0.5
1.0
1.5
2.0
2.5
Fold change
B
Time (min)
0
90
120
180
CHX
Puromycin
ACTIN
IGF2BP3
CHX
SEM NT
SEM I3sg2
ns
Fold Change
SEM NT2
SEM I3sg5
ns
Fold Change
C
PKM RQ Compared to Cyto
SEM NT
SEM I3sg2
Fractions
PKM RQC ompared to Cyto
(-) EDTA
(+) EDTA
Fractions
D
PSAT1 RQ Compared to Cyto
SEM NT
SEM I3sg2
Fractions
PSAT1 RQ Compared to Cyto
(-) EDTA
(+) EDTA
Fractions
E
SHMT1 RQ Compared to Cyto
SEM NT
SEM I3sg2
Fractions
SHMT1 RQC ompared to Cyto
(-) EDTA
(+) EDTA
Fractions
F
MTHFR RQ Compared to Cyto
SEM NT
SEM I3sg2
Fractions
MTHFR RQ Compared to Cyto
(-) EDTA
(+) EDTA
Fractions
G
Absorbance (260 nm)
80S
40S
60S
Polysomes
NT
I3sg2
10% Sucrose gradient 45%
Absorbance (260nm)
NT+EDTA
40S
60S
10% Sucrose gradient 45%

Figure S5

A

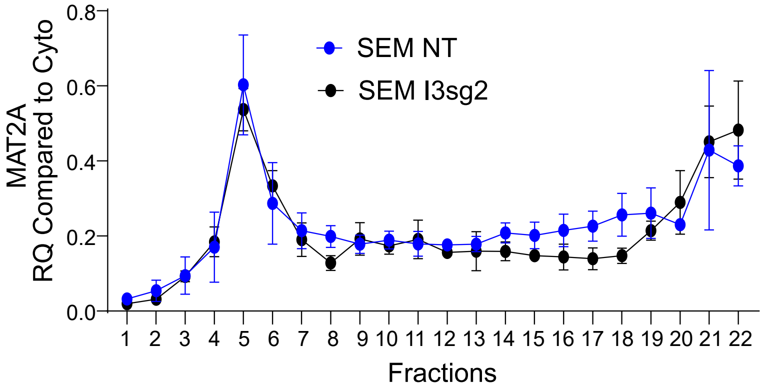

B

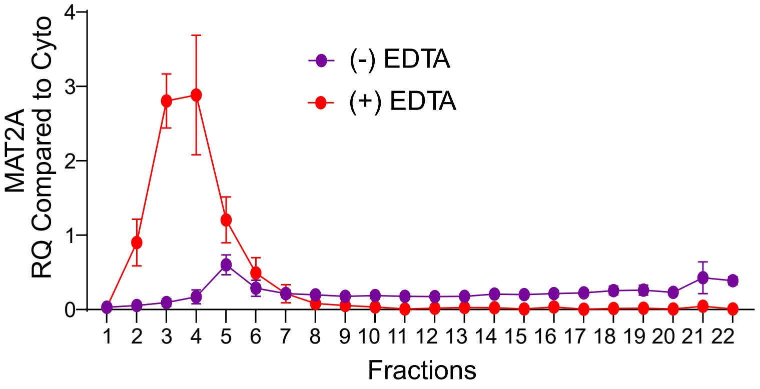

C

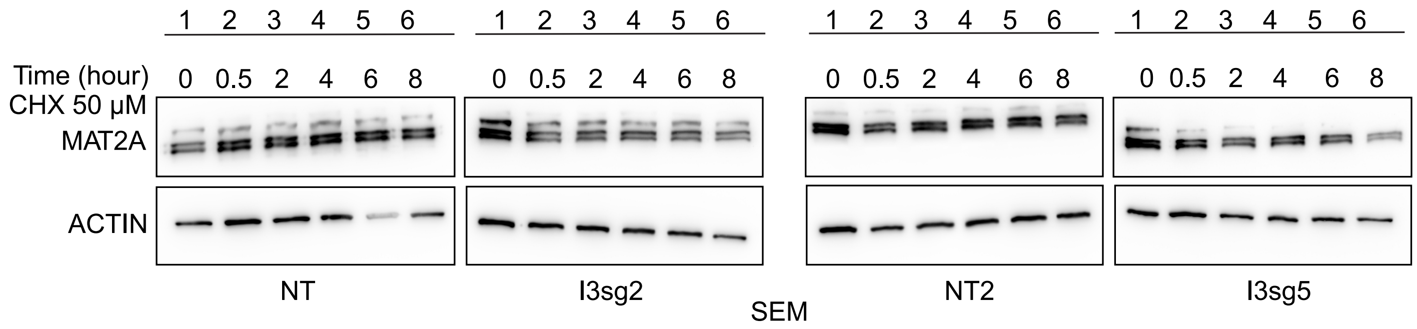

	NT	I3sg2
p-value	0.9882	0.0503
Equation	Y = -0.0006910*X + 1.426	Y = -0.05421*X + 0.7935

	NT2	I3sg5
p-value	0.2943	0.0434
Equation	Y = -0.02492*X + 0.8185	Y = -0.06802*X + 0.7758

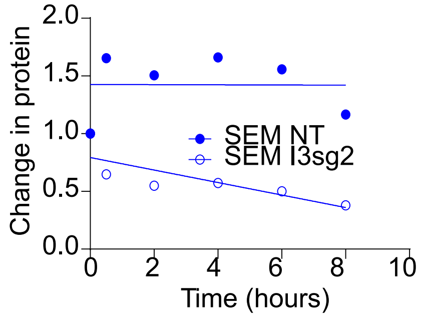

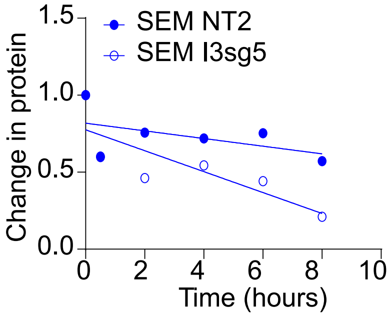

Figure S6

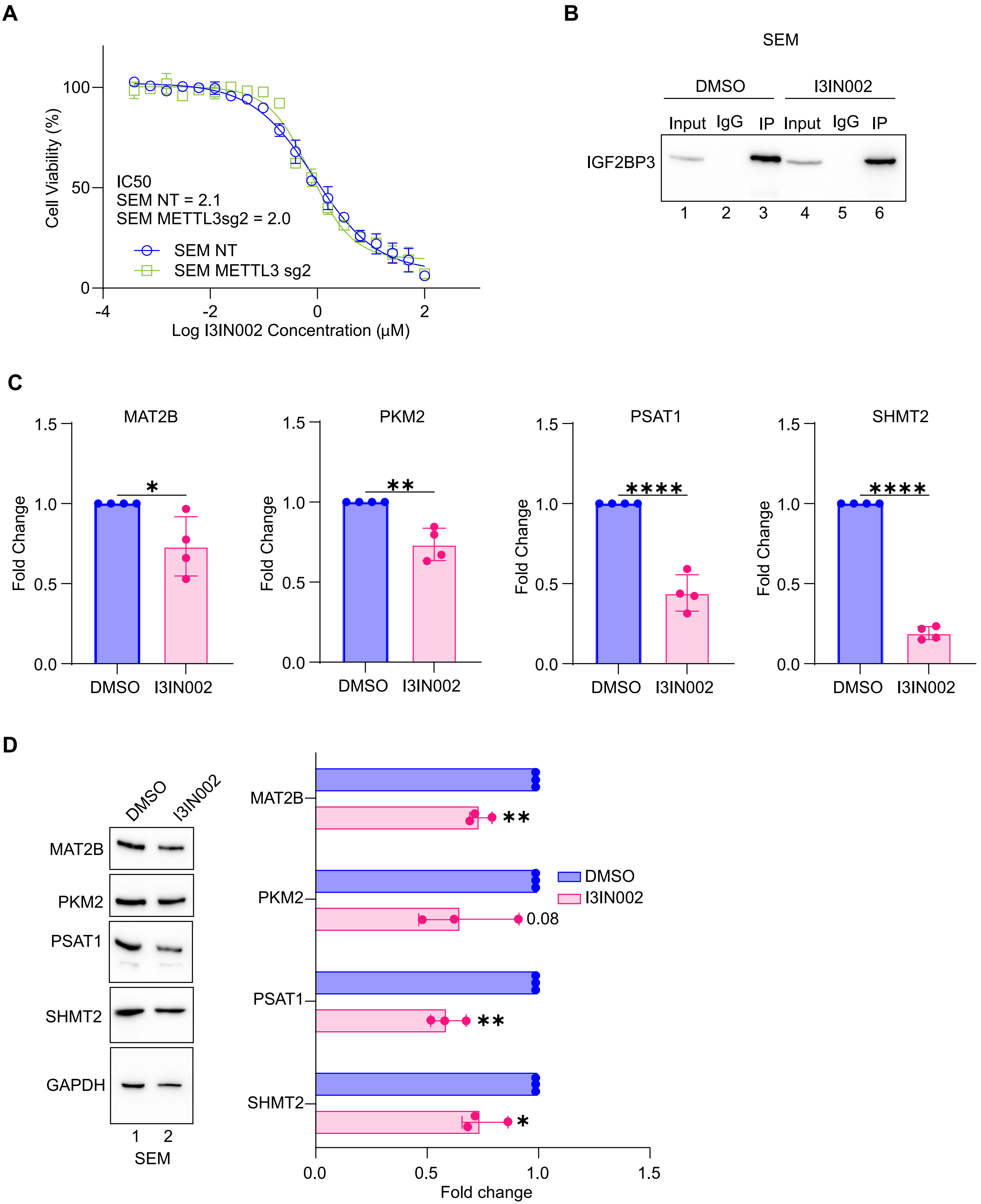
A
Cell Viability (%)
100
50
0
-4
-2
0
2
Log I3IN002 Concentration (µM)
IC50
SEM NT = 2.1
SEM METTL3sg2 = 2.0
SEM NT
SEM METTL3 sg2
B
SEM
DMSO
I3IN002
Input
IgG
IP
Input
IgG
IP
IGF2BP3
1
2
3
4
5
6
C
MAT2B
PKM2
PSAT1
SHMT2
Fold Change
1.5
1.0
0.5
0.0
*
**
****
****
DMSO
I3IN002
D
DMSO
I3IN002
MAT2B
PKM2
PSAT1
SHMT2
GAPDH
1
2
SEM
**
0.08
**
*
DMSO
I3IN002
0.0
0.5
1.0
1.5
Fold change

Figure S7

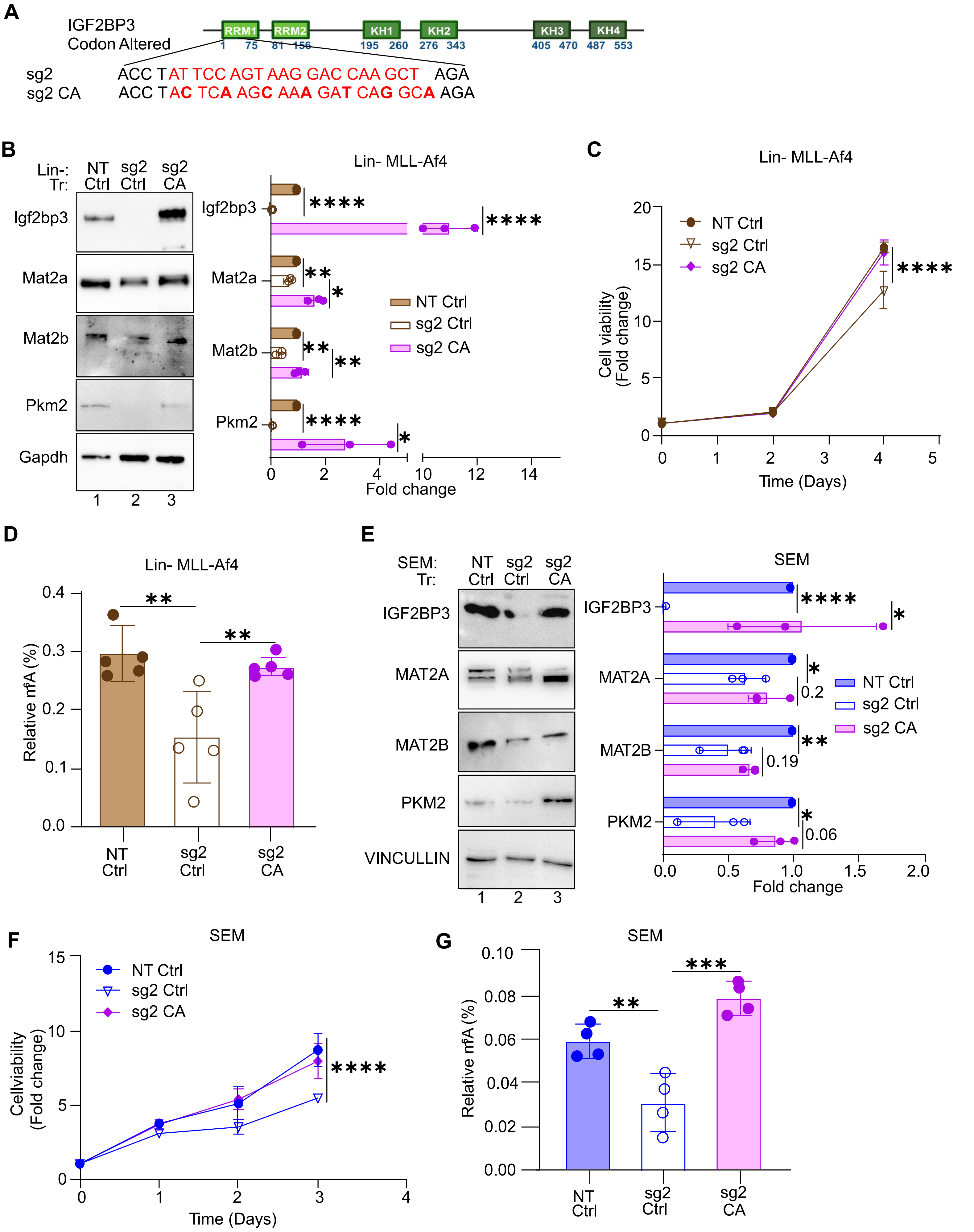
A
IGF2BP3
Codon Altered
RRM1
RRM2
KH1
KH2
KH3
KH4
1 75 81 156 195 260 276 343 405 470 487 553
sg2 ACC TAT TCC AGT AAG GAC CAA GCT AGA
sg2 CA ACC TAC TCA AGC AAA GAT CAG GCA AGA
B
Lin-: NT Ctrl sg2 Ctrl sg2 CA
Tr:
Igf2bp3
Mat2a
Mat2b
Pkm2
Gapdh
1 2 3
Lin- MLL-Af4
NT Ctrl
sg2 Ctrl
sg2 CA
Fold change
C
Lin- MLL-Af4
Cell viability (Fold change)
Time (Days)
D
Lin- MLL-Af4
Relative m6A (%)
E
SEM: NT Ctrl sg2 Ctrl sg2 CA
Tr:
IGF2BP3
MAT2A
MAT2B
PKM2
VINCULLIN
SEM
0.2
0.19
0.06
Fold change
F
SEM
Cellviability (Fold change)
Time (Days)
G
SEM
Relative m6A (%)

Figure S8

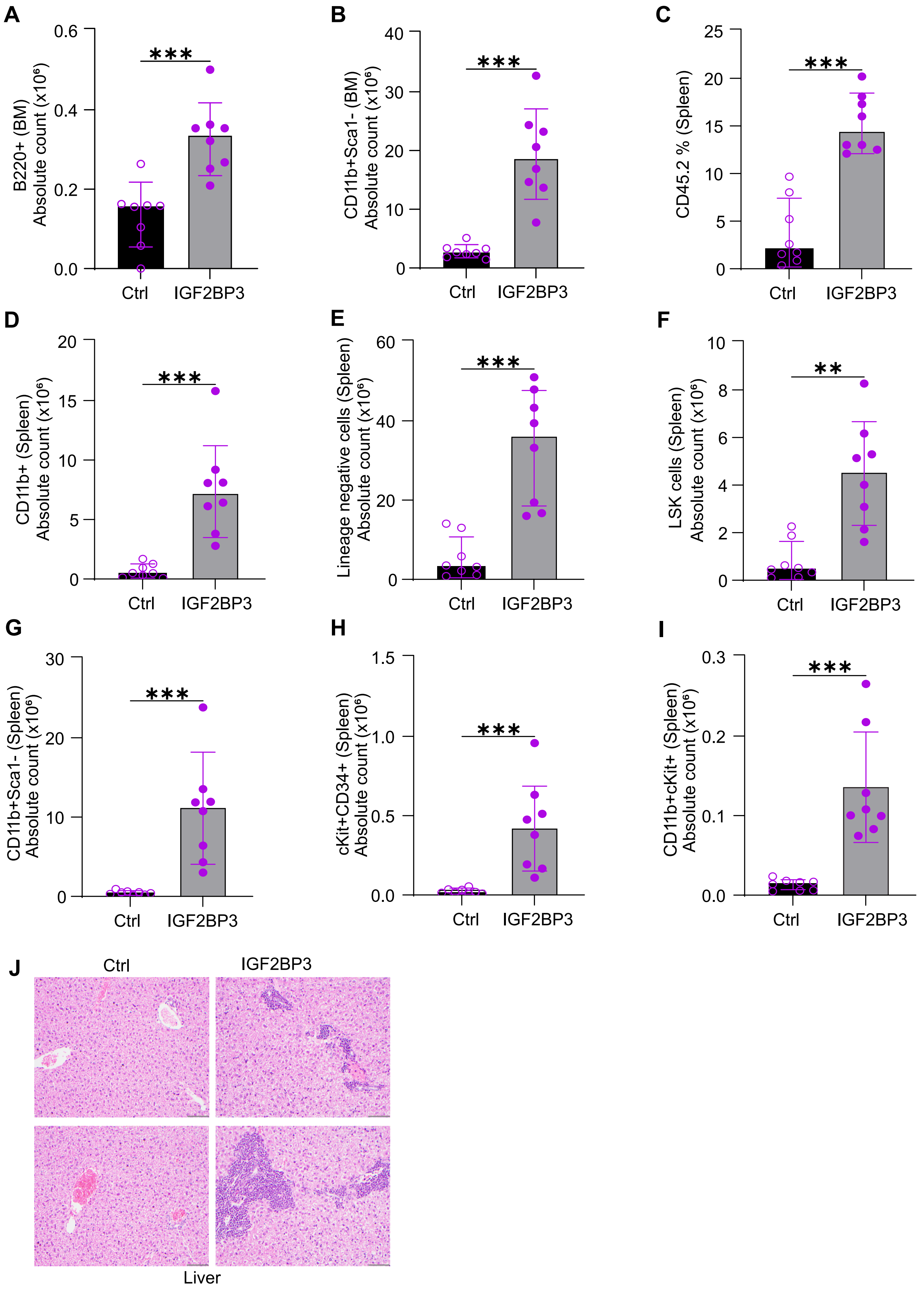

A
B220+ (BM)
Absolute count (x10⁶)
0.6
0.4
0.2
0.0
***
Ctrl
IGF2BP3
B
CD11b+Sca1- (BM)
Absolute count (x10⁶)
40
30
20
10
0
***
Ctrl
IGF2BP3
C
CD45.2 % (Spleen)
25
20
15
10
5
0
***
Ctrl
IGF2BP3
D
CD11b+ (Spleen)
Absolute count (x10⁶)
20
15
10
5
0
***
Ctrl
IGF2BP3
E
Lineage negative cells (Spleen)
Absolute count (x10⁶)
60
40
20
0
***
Ctrl
IGF2BP3
F
LSK cells (Spleen)
Absolute count (x10⁶)
10
8
6
4
2
0
**
Ctrl
IGF2BP3
G
CD11b+Sca1- (Spleen)
Absolute count (x10⁶)
30
20
10
0
***
Ctrl
IGF2BP3
H
cKit+CD34+ (Spleen)
Absolute count (x10⁶)
1.5
1.0
0.5
0.0
***
Ctrl
IGF2BP3
I
CD11b+cKit+ (Spleen)
Absolute count (x10⁶)
0.3
0.2
0.1
0.0
***
Ctrl
IGF2BP3
J
Ctrl
IGF2BP3
Liver

SUPPLEMENTARY FIGURE LEGENDS

Figure S1. IGF2BP3 does not grossly regulate oxidative phosphorylation in B-ALL cells.

A. Seahorse XF kinetic trace for Oxygen consumption rate (OCR) for control versus IGF2BP3-depleted SEM cells.

B. Maximal respiration rate measurements for control versus IGF2BP3-depleted SEM cells, as measured in Seahorse experiments.

C. Rate of ATP generation from oxidative phosphorylation for control versus IGF2BP3-depleted SEM cells, as measured in Seahorse experiments.

D. ATP-linked respiration measurement for control versus IGF2BP3-depleted NALM6 cells, as measured in Seahorse experiments.

E. Maximal respiration measurements for control versus IGF2BP3-depleted NALM6 cells, as measured in Seahorse experiments.

F. Rate of ATP generation from oxidative phosphorylation for control versus IGF2BP3-depleted NALM6 cells, as measured in Seahorse experiments.

G. Steady-state levels of TCA-cycle intermediates measured by GC/MS in control versus IGF2BP3-depleted (I3sg2) SEM cells.

H. Steady-state levels of amino acids measured by GC/MS in control versus IGF2BP3-depleted (I3sg2) SEM cells.

I. Incorporation of carbon from ^{13}C-labeled glucose, into citric acid cycle intermediates (citrate, alpha-ketoglutarate, fumarate and malate) measured as mole percent enrichment (MPE) from GC-MS experiments.

J. Incorporation of carbon from ^{13}C-labeled glutamine into citric acid cycle intermediates (citrate, alpha-ketoglutarate, fumarate and malate), measured as mole percent enrichment (MPE) from GC-MS experiments.

All data are n ≥ 4 replicates represented as mean ± standard deviation (SD), compared by two-sided unpaired t-test; *, p<0.05; **, p<0.01; ***, p<0.001. All experiments were repeated at least twice for consistency.

Figure S2. Additional metabolites show consistent changes in both knockout lines of IGF2BP3.

A-B. Abundance of Lactate and Fructose-1,6-bisphosphate measured by LC-MS in control (NT, NT2) versus IGF2BP3-depleted (I3sg2, I3sg5) SEM cells.

C-D. Abundance and incorporation of carbon from ^{13}C-labeled glucose into Cystathionine measured as mole percent enrichment (MPE) from LC-MS experiments.

All data are n = 3 replicates represented as mean ± standard deviation (SD), compared by two-sided unpaired t-test; *, p<0.05; **, p<0.01; ***, p<0.001. All experiments were repeated at least twice for consistency.

Figure S3. Related to Figure 4.

A. ELISA measurement of m^{6}A modification on mRNA isolated from SEM and Lin- MLL-Af4 cells, control or depleted for IGF2BP3; n=4.

B. RNA m^{6}A methylase activity (colorimetric assay, expressed as enzymatic activity) in SEM cells, control or depleted for IGF2BP3; (n=5; n (sg5) = 4).

C. RNA m^{6}A demethylase activity (colorimetric assay, expressed as enzymatic activity) in SEM cells, control or depleted for IGF2BP3; n=5.

D. Western blot analysis of RNA m^{6}A-methylase and demethylase enzymes in SEM cells, control or depleted for IGF2BP3; n=3.

E. Metagene plots depicting the changes in the m^{6}A peak coverage and IGF2BP3 binding coverage spanning a region of ± 400 bp from the stop codon in SEM control and IGF2BP3-depleted cells (top), along with their respective motifs (bottom).

F. Change in m^{6}A peak counts across different transcripts in SEM control and IGF2BP3-depleted cells.

G. Heatmap showing the transcripts which lost or gained m^{6}A peak(s) in SEM control and IGF2BP3-depleted cells.

H. Enrichment analysis for metabolism-specific pathways (top) with Human Cyc module and GO Biological Processes (down) corresponding to the genes mentioned above using Enrichr.

All data replicates represented as mean ± standard deviation (SD), compared by two-sided unpaired t-test; *, p<0.05; **, p<0.01; ***, p<0.001. In case of missing or outlier values (in cases where no signal was detected or the signal exceeded the instrument's detection limit), the replicate was not reported. All experiments were repeated at least twice for consistency. All western blots were repeated at least three times to report the changes, if any.

Figure S4. Related to Figure 4.

A. Western blot analysis of key metabolic enzymes and m^{6}A machinery genes in Lin- MLL-Af4 cells. Numbers alongside the bars represent p-values.

B. Western blot analysis of puromycin incorporation for studying changes in the global translation at different time points (SuNSET Assay) in IGF2BP3 expressing and depleted cells (left: sg2; right sg5). β-Actin (ACTIN) was used as a loading control.

C-F. As in F, 10-45% Sucrose gradient fractionation of cytosolic extracts from control or IGF2BP3-depleted SEM cells (left) along with the respective EDTA control (right). mRNA distribution of PKM, PSAT1, SHMT1, and MTHFR mRNAs was measured by RT-qPCR (IGF2BP3 targets that showed increases or mild decreases in protein expression levels). Represented as mean ± standard deviation (SD).

G. A_{260} absorbance curve for the polysome profiling data along with the EDTA control.

All data are n = 3 replicates. All data represented as mean ± standard deviation (SD), compared by two-sided unpaired t-test; *, $p<0.05$; **, $p<0.01$; ***, $p<0.001$. p-values were reported where a subtle change was visualized at the protein levels. All experiments were repeated at least twice for consistency. All the western blots were repeated at least three times to report the changes, if any.

Figure S5. Related to Figure 4.

A.-B.10-45% Sucrose gradient fractionation of cytosolic extracts from control or IGF2BP3-depleted SEM cells, along with the EDTA control. MAT2A mRNA distribution was measured by RT-qPCR.

C. Western blot analysis of the Cycloheximide Chase Assay in IGF2BP3 sufficient and depleted cells (left: I3sg2; right: I3sg5) for MAT2A. β-Actin (ACTIN) was used as a loading control. ImageJ software was used to quantify the change in protein amounts of MAT2A over time and plotted as a graph in terms of fold change (IGF2BP3-depleted/NT). For quantification, 0-hour values were set to 1, and the respective changes in the protein levels were calculated by dividing the values of different time points by the 0-hour values. The trendlines illustrate the changes in protein levels, calculated using a linear regression model.

All data are n = 3 replicates. All experiments were repeated at least twice for consistency. All the western blots were repeated at least three times to report the changes, if any.

Figure S6. Related to Figure 5.

A. Cell viability assays (Cell Titer Glo) on IGF2BP3 deleted SEM vs METTL3-knockdown SEM cells treated with I3IN-002. Cells were grown for 3 days in the presence of the inhibitor before measuring cell viability. Viability has been normalized to their respective DMSO control-treated cells; mean ± standard deviation (SD); n = 5.

B. Western blot analysis of IGF2BP3 pull-down after immunoprecipitation in the DMSO-treated and I3IN-002-treated SEM cells. Crude lysate is represented by “Input”, IgG is the negative control for the IP.

C. RT-qPCR of IGF2BP3 target transcripts from the RNA isolated after IGF2BP3 pull down from the DMSO-treated and I3IN-002-treated cells; n=4.

D. Western blot analysis of IGF2BP3 targets from the immunoprecipitated lysates enriched after IGF2BP3 pull down from the DMSO-treated and I3IN-002-treated cells. Numbers alongside the bars represent p-values; n=3.

All data replicates represented as mean ± standard deviation (SD), compared by two-sided unpaired t-test; *, $p<0.05$; **, $p<0.01$; ***, $p<0.001$. All experiments were repeated at least twice for consistency. All the western blots were repeated at least three times to report the changes, if any.

Figure S7. Related to Figure 6.

A. MSCV-based construct showing bases altered to render it insensitive to sg2-mediated CRISPR/Cas9 activity (“codon-altered”, CA).

B. Western blot analysis of enforced expression of IGF2BP3 in Lin- MLL-Af4 cells that were previously depleted for IGF2BP3. NT/Ctrl, Lin- MLL-Af4 cells sufficient for IGF2BP3, transduced with control vector; sg2/MIG, Lin-

MLL-Af4 cells depleted for IGF2BP3, transduced with control vector; sg2/CA, Lin- MLL-Af4 cells depleted for IGF2BP3, then transduced with codon-altered IGF2BP3. Additionally, Western blot analysis for PKM2, MAT2A, and MAT2B in Lin- MLL-Af4 cells is shown. Numbers alongside the bars represent p-values; n=3.

C. Cell growth curves measured by Cell Titer Glo, over three days in Lin- MLL-Af4 cells, notated as in (B). Viability has been normalized to control cells; mean ± standard deviation (SD); n = 5, one-way ANOVA followed by Bonferroni's multiple comparisons test; ****P < 0.0001.

D. ELISA measurement of m^6A modification in RNA isolated from Lin- MLL-Af4 cells notated as in (B); reported as mean ± standard deviation (SD) by two-sided unpaired t-test; **, p<0.01, ***, p<0.001; n = 4.

E. Western blot analysis of enforced expression of IGF2BP3 in SEM cells that were previously depleted for IGF2BP3. NT/Ctrl, SEM cells sufficient for IGF2BP3, transduced with control vector; sg2/MIG, SEM cells depleted for IGF2BP3, transduced with control vector; sg2/CA, SEM cells depleted for IGF2BP3 then transduced with codon-altered IGF2BP3. Additionally, Western blot analysis for PKM2, MAT2A, MAT2B in SEM cells is shown. Numbers alongside the bars represent p-values; n=3.

F. Cell growth curves measured by Cell Titer Glo over three days in SEM cells, notated as in (C). Viability has been normalized to control cells; mean ± standard deviation (SD); n = 5, one-way ANOVA followed by Bonferroni's multiple comparisons test; ****P < 0.0001.

G. ELISA measurement of m^6A modification in RNA isolated from SEM cells notated as in (B); reported as mean ± standard deviation (SD) by two-sided unpaired t-test; **, p<0.01, ***, p<0.001; n = 4.

All data replicates represented as mean ± standard deviation (SD). All experiments were repeated at least twice for consistency. All the western blots were repeated at least three times to report the changes, if any.

Figure S8. Related to Figure 6.

A. Quantitation of bone marrow B220+ cell count in mice transplanted with MLL-Af4 re-expressing empty vector (Ctrl) or IGF2BP3 in the two groups at 6 weeks.

B. Quantitation of bone marrow CD11b+Sca1- cell count in mice transplanted with MLL-Af4 re-expressing empty vector (Ctrl) or IGF2BP3 in the two groups at 6 weeks.

C. Percentage engraftment of CD45.2 Lin- cells in spleen from *Igf2bp3*$^{del/del}$ mice transduced with MLL-Af4 re-expressing empty vector (Ctrl) or IGF2BP3 in the two groups at 6 weeks.

D. Quantitation of splenic CD11b+ cell count in mice transplanted with MLL-Af4 re-expressing empty vector (Ctrl) or IGF2BP3 in the two groups at 6 weeks.

E. Quantitation of splenic lineage-negative cell count along with representative FACS plots in mice transplanted with MLL-Af4 re-expressing empty vector (Ctrl) or IGF2BP3 in the two groups at 6 weeks.

F. Quantitation of splenic LSK (Lin-cKit+Sca1-) cell count in mice transplanted with MLL-Af4 re-expressing empty vector (Ctrl) or IGF2BP3 in the two groups at 6 weeks.

G. Quantitation of splenic CD11b+Sca1- (potential LIC; [S1]) cell count in mice transplanted with MLL-Af4 re-expressing empty vector (Ctrl) or IGF2BP3 in the two groups at 6 weeks.

H. Quantitation of splenic cKit+CD34+ (potential LIC; [S2]) cell count along with representative FACS in mice transplanted with MLL-Af4 re-expressing empty vector (Ctrl) or IGF2BP3 in the two groups at 6 weeks.

I. Quantitation of splenic CD11b+cKit+ (potential LIC; [S2]) cell count along with representative FACS in mice transplanted with MLL-Af4 re-expressing empty vector (Ctrl) or IGF2BP3 in the two groups at 6 weeks.

J. H&E staining of liver of mice transplanted with mice transplanted with MLL-Af4 re-expressing empty vector (Ctrl) or IGF2BP3 in the two groups at 6 weeks. Scale bar: 20X

All data replicates with 8 mice per group represented as mean ± standard deviation (SD), compared by two-sided unpaired t-test; *, p<0.05; **, p<0.01; ***, p<0.001. All experiments were repeated at least twice for consistency.

Supplemental References

S1. Tran TM, Philipp J, Bassi JS, Nibber N, Draper JM, Lin TL, Palanichamy JK, Jaiswal AK, Silva O, Paing M, et al. (2022). The RNA-binding protein IGF2BP3 is critical for MLL-AF4-mediated leukemogenesis. *Leukemia*. 36(1):68-79. doi:10.1038/s41375-021-01346-7

S2. Lin TL, Jaiswal AK, Ritter AJ, Reppas J, Tran TM, Neeb ZT, Katzman S, Thaxton ML, Cohen A, Sanford JR, et al. (2024). Targeting IGF2BP3 enhances antileukemic effects of menin-MLL inhibition in MLL-AF4 leukemia. *Blood Adv*. 8(2):261-275. doi:10.1182/bloodadvances.2023011132

TABLE S2: List of qPCR primer sequences used in this study

Polysome Fractionation

NAME	SEQUENCE
MAT2A F	GACCAGGGCTTAATGTTTGGC
MAT2A R	TAGAATCAGGGCGTAACCAAGG
MAT2B F	GAGAACAATCTAGGAGCTGCTG
MAT2B R	TGCCAGTGATCCATGTTTGC
MTHFR F	ATCTGTGTGGCAGGTTACCC
MTHFR R	AAGCGGAAGAATGTGTCAGC
PKM F	TGTACCATTGGCCCAGCTTC
PKM R	TGTGCGCACATTCTTGATGG
PSAT1 F	TCCATTCCGCATTGGCAATG
PSAT1 R	ATGCCTCCCACAGACCTATG
SHMT1 F	AGTCACAGGTGGTTCTGACAAC
SHMT1 R	AACAGGCTTCTAGCACCTTCTC

RIP-qPCR

NAME	SEQUENCE
PKM2 F	GTACCGTGGCATCTTCCCTG
PKM2 R	CATCACGGCACAGGAACAAC
MAT2B F	TAGGAGCTGCTGTTTTGAGGA
MAT2B R	GCTGCCAGTGATCCATGTTT
PSAT1 F	TTTTCTGCAAGGAGGTGGGT
PSAT1 R	CTCCCAAGTTTAGGGTGAACGA
SHMT2 F	GGGCCTTGGAAGCCTTTGAC
SHMT2 R	CATGATCCGGTCGTGAGGT
18S F	GAGGATGAGGTGGAACGTGT
18S R	GGACCTGGCTGTATTTTCCA
